# First-trimester nonsteroidal anti-inflammatory drugs exposure and risk of major congenital malformations: A retrospective register-based cohort study

**DOI:** 10.1371/journal.pmed.1005063

**Published:** 2026-05-14

**Authors:** Ariel Avraham Hasidim, Itamar Ben Shitrit, Daphna Idan, Tal Michael, Amalia Levy, Gali Pariente, Eitan Lunenfeld, Sharon Daniel

**Affiliations:** 1 Department of Epidemiology, Biostatistics, and Community Health Sciences, School of Public Health, Faculty of Health Sciences, Ben-Gurion University of the Negev, Beer-Sheva, Israel; 2 Department of Pediatrics A, Schneider Children’s Medical Center of Israel, Petah Tikva, Israel; 3 Sackler Faculty of Medicine, Tel Aviv University, Tel Aviv, Israel; 4 Joyce and Irving Goldman Medical School, Faculty of Health Sciences, Ben-Gurion University of the Negev, Beer-Sheva, Israel; 5 Clinical Research Center, Faculty of Health Sciences, Soroka University Medical Center, Ben-Gurion University of the Negev, Beer-Sheva, Israel; 6 Department of Obstetrics and Gynecology, Faculty of Health Sciences, Ben-Gurion University of the Negev and Soroka University Medical Center, Beer-Sheva, Israel; 7 Adelson School of Medicine, Ariel University, Ariel, Israel; 8 Department of Pediatrics, Faculty of Health Sciences, Ben-Gurion University of the Negev, Beer-Sheva, Israel; 9 Clalit Health Services, Southern District, Beer-Sheva, Israel; London School of Hygiene and Tropical Medicine, UNITED KINGDOM OF GREAT BRITAIN AND NORTHERN IRELAND

## Abstract

**Background:**

Pain and fever are common in early pregnancy, yet their management poses a major clinical dilemma. Although not confirmed, recent studies have raised safety concerns regarding acetaminophen. Evidence on the use of nonsteroidal anti-inflammatory drugs (NSAID) in the first trimester remains inconclusive. This uncertainty has left clinicians with limited evidence to guide treatment decisions. This study evaluated the association between first-trimester NSAID exposure and the risk of major congenital malformations (MCMs) in a large, population-based cohort of pregnancies.

**Methods and findings:**

We conducted a population-based retrospective cohort study within the Southern Israeli Pregnancy Registry (siPREG) project, including all singleton pregnancies of women aged 15–45 years resulting in live births, stillbirths, or elective terminations for fetal malformations at a Soroka University Medical Center between 1998 and 2018. Pregnancies exposed to established teratogens, multiple gestations, and those with documented genetic or chromosomal anomalies were excluded. First-trimester NSAID exposure was defined by pharmacy dispensations (overall and by specific agents). MCMs were identified from linked clinical, hospitalization, and termination records through the first postnatal year.

Propensity scores were estimated using covariates selected via a directed acyclic graph, including maternal age, ethnicity, diabetes, medical indication for NSAID use, exposure to other antipyretics, obesity, smoking, folic-acid use, gravidity, perinatal care, and year of pregnancy. Generalized full matching was used to balance covariates. Adjusted risk ratios were derived using weighted Poisson regression with G-computation, and two-way cluster-robust standard errors, jointly clustering by maternal identifier and matching subclass. Sensitivity analyses included a dose–response assessment across defined-daily-dose (DDD) categories and a tipping-point analysis evaluating the impact of potential misclassification from unrecorded over-the-counter NSAID use.

A total of 264,858 singleton pregnancies were included in the final cohort; 20,202 (7.6%) were exposed to NSAID, most commonly ibuprofen (5.1%), diclofenac (1.6%), and naproxen (1.2%). NSAID exposure, in total and as individual agents, was not associated with MCMs overall (8.2% versus 7.0%; matched-adjusted-Relative Risk (aRR) = 0.99 (95% CI [0.90,1.10])) or with organ-system-specific MCMs, including cardiovascular (matched-aRR = 1.05 (95% CI [0.92,1.20]), musculoskeletal (matched-aRR = 1.03 (95% CI [0.77,1.39])), central nervous system (matched-aRR = 0.77 (95% CI [0.53,1.11])), cleft palate (matched-aRR = 0.95 (95% CI [0.47–1.91])), gastrointestinal (matched-aRR = 1.03 (95% CI [0.64–1.63])), and genitourinary (matched-aRR = 0.99 (95% CI [0.72,1.35])) malformations. Dose–response analyses showed no significant association with MCMs across cumulative NSAID exposure: short-term (1–7 DDD, matched-aRR = 1.06 (95% CI [0.97,1.15]), medium-term (8–21 DDD, matched-aRR = 1.10 (95% CI [0.99,1.22]), and long-term (>21 DDD, matched-aRR = 1.24 (95% CI [0.94,1.63])). The main limitation was the potential for minor exposure misclassification due to over-the-counter availability of ibuprofen, although sensitivity analyses simulating such misclassification suggested minimal impact on the risk estimates.

**Conclusion:**

In this large, population-based cohort, we found no evidence supporting an association between first-trimester exposure to NSAID and MCMs, providing reassuring evidence regarding their fetal safety in early pregnancy.

## Author summary

**Why was this study done?:**

- Nonsteroidal anti-inflammatory drugs (NSAID) are medications commonly used to reduce pain, inflammation, and fever, and include drugs such as ibuprofen, diclofenac, and naproxen.
- Pain and fever are common in early pregnancy, but evidence on the safety of commonly used pain relievers, including NSAID, is limited.
- Previous studies raised concerns about some medications, but it remained unclear whether NSAID in the first trimester increase the risk of major birth defects.

**What did the researchers do and find?:**

- We analyzed 264,858 pregnancies from a large population-based registry in Southern Israel, including over 20,000 pregnancies exposed to NSAID in the first trimester.
- We compared the risk of major birth defects between exposed and unexposed pregnancies, adjusting for maternal and pregnancy characteristics.
- We found no increased risk of overall or organ-specific birth defects associated with NSAID use, including ibuprofen, diclofenac, or naproxen.

**What do these findings mean?:**

- Our results provide reassuring evidence that NSAID use in early pregnancy is not associated with major birth defects.
- These findings can help both pregnant women and physicians make informed decisions about managing pain and fever in early pregnancy.
- One limitation of this study is that some ibuprofen use may not have been recorded, as it can be purchased over the counter. However, additional analyses suggest that any missing information is unlikely to have meaningfully affected the results.

## Abstract 3

In a retrospective register-based study, Ariel Hasidim, Itamar Shitrit and colleagues investigate the association between first trimester anti-inflammatory drug exposure and major congenital malformations, in a large Israeli cohort.

## Introduction

During September 2025 regulatory advisories in the United States have raised concerns about potential adverse pregnancy outcomes associated with acetaminophen use during gestation [1]. Evidence suggest that a considerable number of pregnant women already avoid the use of analgesics due to concerns about fetal safety [2]. However, Inadequate analgetic management can cause significant maternal distress, increase the risk of perinatal depression, and potentially impair neonatal development [3,4].

In addition to pain relief, the management of fever represents another important clinical challenge during pregnancy. Untreated maternal fever, particularly during the first trimester, has been linked to an increased risk of adverse fetal outcomes, including neural tube defects, congenital heart defects, oral clefts, and neurodevelopmental disorders [5–8]. In this context, the growing concerns about the safety of commonly used analgesic and antipyretic medications may leave pregnant individuals without effective options for managing pain and fever, potentially exposing them to the risks associated with leaving these conditions untreated.

Among the most commonly used medications with analgesic and antipyretic properties are nonsteroidal anti-inflammatory drugs (NSAID), with estimates indicating that 5%–20% of women report their use during early pregnancy [9–12]. The potential association between exposure to NSAID during the first trimester and the risk of major congenital malformations (MCMs) has been previously examined, however findings remain inconsistent. While some studies have reported increased risks of specific adverse outcomes [9,13,14], other large prospective cohort studies have not demonstrated such associations [15,16]. Smaller case-control studies have suggested possible associations with ventricular septal defects [17,18], whereas drug-specific analyses, such as those focusing on diclofenac, have not identified an excess risk [19].

This population-based cohort study was designed to assess the association between first trimester maternal exposure to NSAID, both overall and for specific drugs, and an increased risk of MCMs. We hypothesized that any potential increase in risk would be modest. In light of growing concerns about the safety of analgesic and antipyretic medications during pregnancy, and the potential consequences of leaving pain or fever, untreated, timely evidence on commonly used therapies - based on large, population-based studies with methodological rigor - is needed.

## Methods

### Study design

This study was conducted within the framework of the Southern Israeli Pregnancy Registry (siPREG), a population-based initiative investigating maternal and perinatal outcomes in southern Israel. This study is reported as per STROBE guideline [20] (STROBE Checklist D1 in S1 File) and was approved by the Soroka University Medical Center (SUMC) ethics committee in accordance with the Declaration of Helsinki (approval number 0069–20-SOR; 7 March 2022). Informed consent was waived by the ethics committee due to the study’s retrospective design.

This retrospective observational study did not follow a prospective analysis plan. Analyses were specified after data extraction based on the study objectives.

### Setting and participants

The cohort included all pregnancies of women aged 15–45 years insured by Clalit Health Services (CHS) maintenance organization in southern Israel that resulted in delivery or elective pregnancy termination for suspected fetal malformations at SUMC between 1998 and 2018. Clalit insures approximately 70% of women of reproductive age in the region, and SUMC accounts for nearly all deliveries (~98%) in the district [21]. Health insurance in Israel is universal and legally mandated. No differences were reported between individuals insured by Clalit and other state-mandated HMOs in the southern district [22].

Pregnancies exposed to established teratogenic drugs (antimetabolites, isotretinoin, and anti-epileptic drugs), multiple gestations, or pregnancies with documented genetic or chromosomal diagnoses were excluded from the study. Analyses were restricted to pregnancies with complete data on model’s covariates and outcomes.

### Exposure

Exposure was defined as dispensation of any NSAID during the first trimester, from the first day of the last menstrual period through the end of the 13th gestational week. Gestational age was assessed by a trained obstetrician, primarily using last menstrual period dates recorded at first-trimester visits, and in a small minority of cases based on first-trimester ultrasound measurements.

In addition, exposure was evaluated separately for each NSAID: ibuprofen, diclofenac, etodolac, naproxen, indomethacin, piroxicam, and lornoxicam. The unexposed group comprised pregnancies unexposed to NSAID during the first trimester, regardless of exposure to other analgesic or antipyretic medications. Pregnancies with exposure to more than one NSAID during the first trimester were included in each relevant drug-specific analysis, such that exposure groups were not mutually exclusive.

Overall exposure was further quantified by the total number of defined daily doses (DDD) dispensed during the first trimester, categorized as no exposure (0 DDD), short-term (1–7 DDD), medium-term (8–21 DDD), and long-term (>21 DDD) exposure. The corresponding DDDs were 1,200 mg for ibuprofen, 100 mg for diclofenac and indomethacin, 400 mg for etodolac, 500 mg for naproxen, 20 mg for piroxicam, and 16 mg for lornoxicam [23].

### Outcomes

MCMs were defined according to the Metropolitan Atlanta Congenital Defects Program (MACDP) of the Centers for Disease Control and Prevention and classified using the International Classification of Diseases, 9th Revision (ICD-9) by board certified neonatologists. We examined associations between first-trimester NSAID exposure and the overall prevalence of MCMs, as well as system-specific groups: cardiovascular (ICD-9: 745, 746, 747), central nervous system (740, 741, 742, 743), musculoskeletal (754, 755, 756), gastrointestinal (750, 751), and genitourinary malformations (752, 753).

### Covariates

Obesity was defined as any diagnosis in the 278.0–278.4 ICD-9 range, diabetes mellitus as ICD-9 250.* and gestational diabetes mellitus (GDM) as ICD-9 648.00–648.04. Folic acid use was defined as dispensation during the first trimester. Smoking was defined based on self-report or ICD-9 code 305.1.

NSAID indications were defined using ICD-9 diagnostic codes recorded during the first trimester of pregnancy, corresponding to conditions for which NSAID are commonly prescribed. These included musculoskeletal and joint disorders (e.g., arthritis, back pain, myalgia; ICD-9 714–716, 718–719, 724, 729), pain and inflammatory conditions (e.g., headache, abdominal or pelvic pain; ICD-9 346, 784, 789, 623–625), injuries and fractures (ICD-9,800–829, 840–848, 920–924, E-codes E931–E980), pregnancy-related conditions including threatened abortion (ICD-9 632), and fever or infection (ICD-9 780.6, 780.60; respiratory/ear, nose, and throat: 460–466, 381*, 462–463; urinary tract: 5,990, 590*; other infections/inflammation: 041*, 079*, 682*, 614*, 615*). A woman could have more than one NSAID indication, and all relevant first-trimester diagnoses were included in the composite variable.

### Dataset assembly

The cohort was constructed by linking four databases. Pregnancy and delivery data were obtained from the SUMC Obstetrics and Gynecology Division, and MCMs diagnosed within the first year of life from the SUMC hospitalization database. Malformations identified before elective terminations were manually retrieved from the SUMC Committee for Termination of Pregnancies registry. All diagnoses were confirmed by board-certified specialists and coded using ICD-9. Medication dispensation data, including prescription and over-the-counter NSAID with Anatomical Therapeutic Chemical classification system (ATC) codes and DDD, were extracted from the CHS database. Databases were merged via national identification number and hospitalization number to link maternal, neonatal, and termination records (Appendix A1 in S1 File).

### Statistical analysis

Statistical analyses were performed using R version 4.5.1 [24]. Categorical variables are reported as *n* (%) and compared using *χ*² tests or Fisher’s exact tests. Continuous variables are summarized as mean ± standard deviation or median [Interquartile Range (IQR)], according to distribution, and compared with two-sample t-tests or Wilcoxon rank-sum tests.

Unmatched-adjusted-Relative Risks (aRRs) were estimated using Poisson regression with a logarithmic link function. Marginal (average) effects were derived with G-computation, and robust variance estimates were obtained with cluster-robust heteroskedasticity-consistent (“sandwich”) estimator clustered by patient identifier [25–28]. Analyses were conducted using the marginaleffects package in R [29].

For matching-adjusted models, generalized full matching [30] was applied on the propensity score to further account for confounding, targeting the average treatment effect (ATE) [31]. The propensity score was estimated using a probit regression of the treatment on the covariates and achieved adequate balance. Matching weights were derived from this procedure and incorporated into the outcome model (no units were discarded by the matching). Matched-aRRs were then estimated using G-computation with weighted Poisson-regression and two-way cluster-robust standard errors, clustered on maternal identifier and matching subclass.

**Fig 1:**
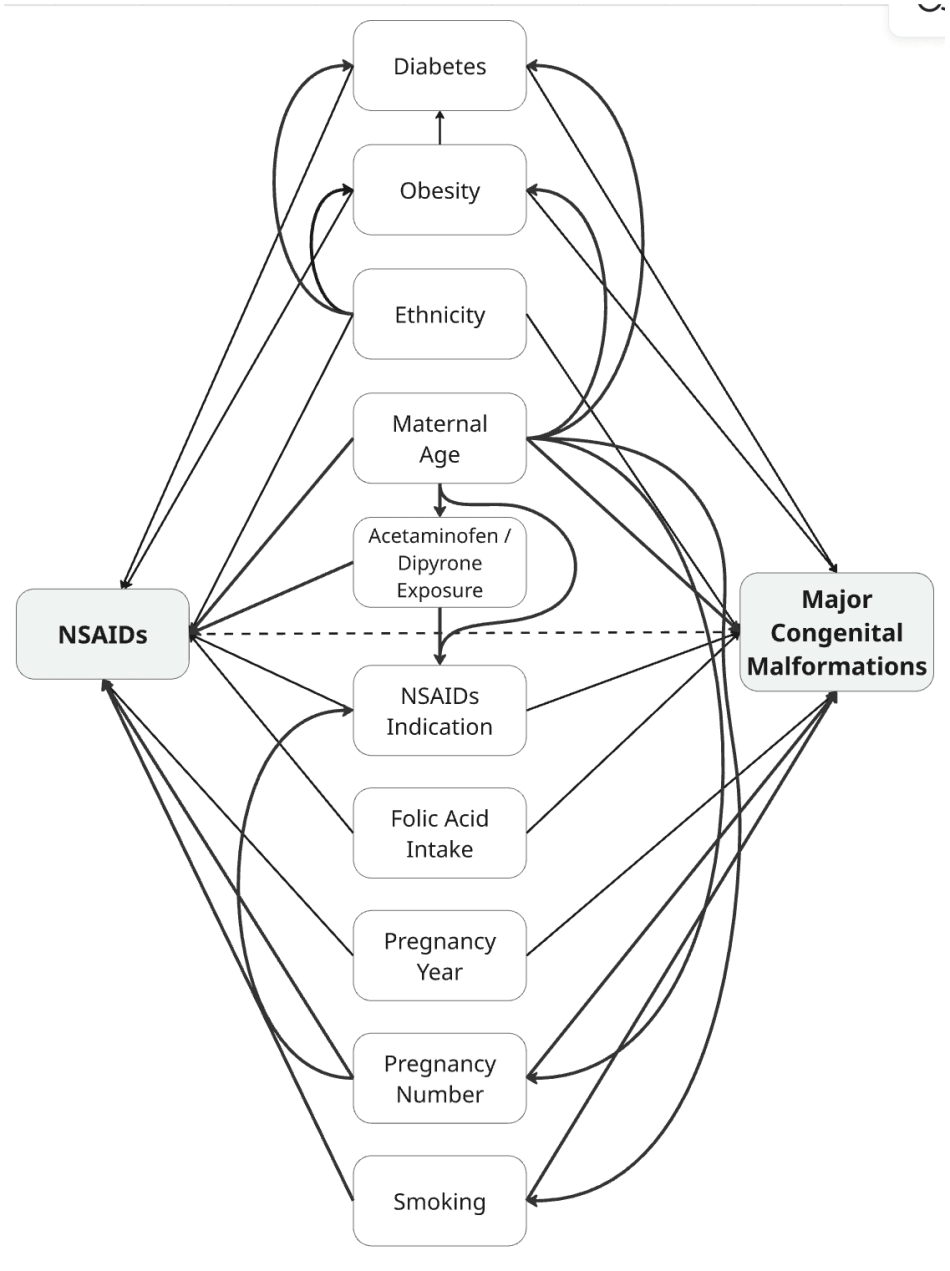
Directed acyclic graph of the assumptions on relationships between variables. The model represents the hypothesized associations between first-trimester NSAID exposure and major congenital malformations. Covariates include maternal factors (age, ethnicity, obesity, diabetes), pregnancy-related variables (number of pregnancies, pregnancy year, indication for NSAID use, and folic acid intake), and other potential confounders that may influence both exposure and outcome. Solid arrows denote assumed direct associations, and the dashed arrow indicates the primary exposure–outcome pathway.

The conceptual model connecting consistent first-trimester NSAID exposure with MCMs is depicted in the directed acyclic graph (DAG), Fig 1. Covariates accounted for in the final models included maternal age, ethnicity, lack of perinatal care, diabetes, obesity, folic acid supplementation, gravidity, calendar year, smoking, NSAID indication, and exposure to other analgesics or antipyretics. These were selected a priori by two experts (S.D. and A.A.H.) to block potential confounding via “backdoor” paths. Maternal diabetes and obesity are associated with cardiac malformations [32–34] and higher NSAID use [35,36]. Lack of perinatal care and folic acid supplementation influence both exposure and malformation risk [37,38]. Ethnicity accounts for higher malformation prevalence and greater use of over-the-counter medications among Bedouins [39]. Calendar year adjusts for temporal changes in NSAID dispensations and detection of MCMs due to increasing prenatal ultrasonography. NSAID indication (e.g., fever or inflammatory conditions) was included to control for confounding by underlying conditions [40,41]. The models were additionally adjusted for first-trimester exposure to other analgesics or anti-pyretics (acetaminophen and dipyrone) to account for other underlying indications for NSAID use.

### Bias and sensitivity analyses

To evaluate the potential impact of exposure misclassification due to over-the-counter ibuprofen purchases not captured in the dispensation database, we conducted a probabilistic sensitivity (“tipping-point”) analysis. Assuming dispensation-based exposure ascertainment is highly specific but may be imperfectly sensitive, we treated recorded ibuprofen exposure as a reliable indicator and simulated increasing degrees of missed exposure by randomly reclassifying individuals from the unexposed to the exposed group. To represent a conservative scenario in which misclassification could bias the association away from the null, we constrained the reclassified subset to have the same prevalence of MCMs observed among the recorded exposed group (8.3%). The proportion reclassified ranged from 0% to 3% of the total cohort in 0.05% increments; at each increment, we performed 100 random reallocations and refit the primary matching-adjusted model to obtain a distribution of adjusted risk ratio estimates (Full details are provided in Appendix A2 in S1 File).

The matching procedure and outcome model included NSAID indication to account for the underlying clinical indication. In response to peer review comments, to further characterize the relationship between NSAID exposure and underlying conditions, we compared the distribution of NSAID indication categories between exposed and unexposed pregnancies. In addition, exposure to other analgesic/antipyretic medications (acetaminophen and dipyrone) was incorporated into the matching preprocessing and outcome model as an indirect proxy for NSAID indications. However, because these medications may be used concurrently with NSAID, their inclusion may introduce temporal ambiguity, overadjustment, or collider bias. We therefore performed four additional sensitivity analyses: (1) removing the exposure to other analgesic/antipyretic medications variable from both matching and model adjustment; (2) limiting matching and adjustment to exposures that occurred before NSAID use; (3) limiting matching to exposures that occurred before NSAID use and additionally adjusting in the outcome model for exposures occurring before and after NSAID use as separate covariates; and (4) excluding pregnancies with exposure to other analgesic/antipyretic medications from both the exposed and unexposed groups.

To account for NSAID dispensations that may have started shortly before conception and extended into the first trimester, we conducted another sensitivity analysis redefining first-trimester exposure to include NSAID dispensations initiated within the two weeks preceding the estimated date of conception.

## Results

**Fig 2:**
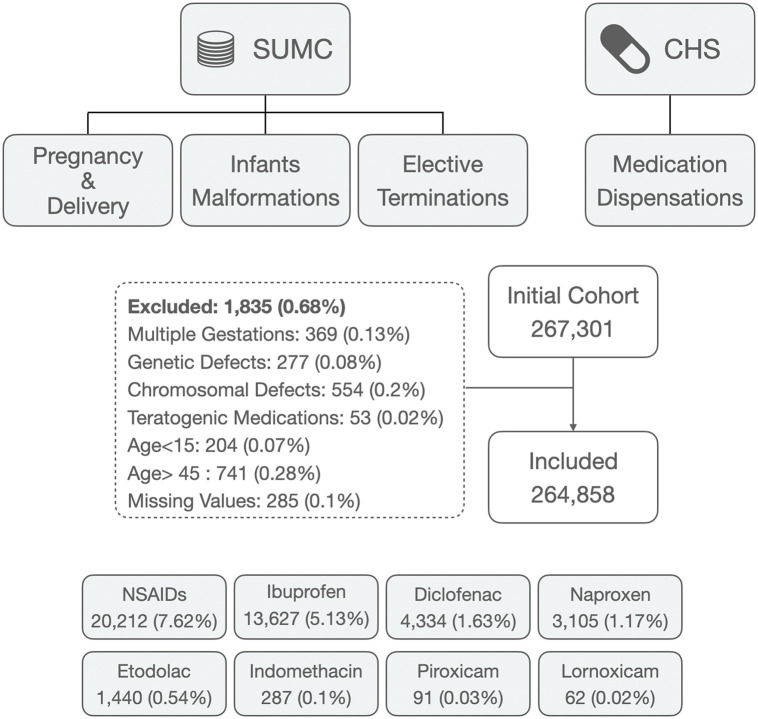
Flow diagram of cohort selection and distribution of first-trimester exposure to individual NSAID. NSAID, NonSteroidal Anti-Inflammatory Drugs.

A total of 267,301 births and elective terminations occurred at SUMC between 1998 and 2018. Of these, 265,143 pregnancies met eligibility criteria, and 264,858 complete cases were included in the final cohort (Fig 2). Among those, 20,202 pregnancies (7.6%) were exposed to NSAID during the first trimester of pregnancy, including ibuprofen (13,627; 5.13%), diclofenac (4,334; 1.63%), naproxen (3,105; 1.17%), etodolac (1,440; 0.54%), indomethacin (287; 0.1%), piroxicam (91; 0.03%), and lornoxicam (62; 0.02%). The patterns of multiple NSAID exposures during the first trimester are presented in Fig A1 in S1 File.

**Fig 3:**
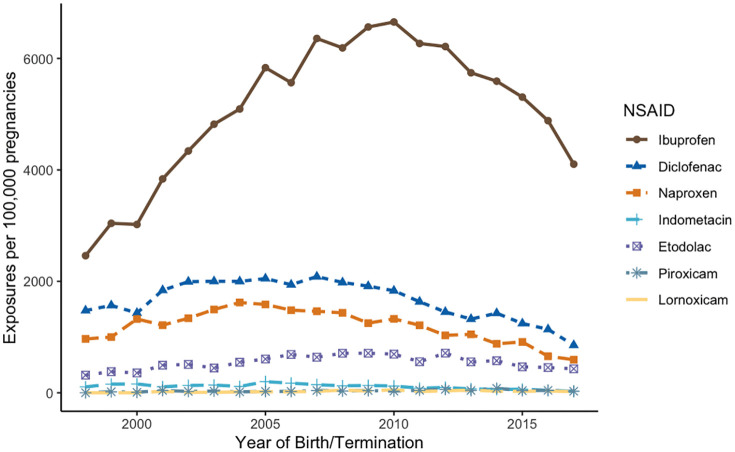
Trends in first-trimester NSAID use by type, 1998–2018. NSAID: NonSteroidal Anti-Inflammatory Drugs. Temporal trends in first-trimester NSAID use among the study cohort. Ibuprofen use increased until 2010 before declining, yet it remained the most commonly dispensed NSAID throughout the study period. Diclofenac and naproxen showed modest increases in the early 2000s followed by a decline, whereas etodolac use rose steadily over time. The data underlying this figure are available as CSV files at: https://github.com/arielhasidim/nsaids-major-malformations-2026.

First-trimester NSAID use increased until 2010, driven primarily by ibuprofen, before declining. Nevertheless, ibuprofen remained the most dispensed NSAID throughout the study period. Diclofenac and naproxen showed modest early-2000s increases before decreasing, while etodolac use rose modestly and steadily over time (Fig 3).

Table 1 presents a comparison of pregnancy and maternal characteristics between pregnancies exposed and unexposed to NSAID during the first trimester. Exposed pregnancies were more often of Bedouin ethnicity (77% versus 53%, Standardized Mean Difference [SMD] = 0.50), had higher gravidity (median 4 (IQR; 2, 6) versus 3 (IQR; 2, 5), with difference of −0.70 with *P*-value < 0.001 and 95%CI [−0.73,-0.67]), higher maternal obesity (1.3% versus 0.5%, SMD = 0.09), and higher folic acid use (45% versus 14%, SMD = 0.72). Clinical indications for which NSAID are commonly prescribed were more prevalent among exposed pregnancies than among unexposed pregnancies (13% versus 9.3%, SMD = 0.13), while pregnancy terminations (0.2% versus 0.6%, SMD = 0.06) were more frequent in the unexposed group.

**Table 1:** Comparison of maternal and pregnancy characteristics between pregnancies exposed and unexposed to NSAID in the first trimester.

Characteristic	NSAID *N* = 20,2,02^1^	Unexposed *N* = 244,6,56^1^	*p*-value^2^	aSMD before	aSMD after
**Calendar year of birth** ^*^			<0.001	0.06	0.03
1998–2002	3,415 (17%)	55,907 (23%)			
2003–2007	5,304 (26%)	56,688 (23%)			
2008–2012	6,105 (30%)	61,087 (25%)			
2013–2017	5,378 (27%)	70,974 (29%)			
**Maternal delivery age, years** ^*^			<0.001	0.06	0.03
<20	462 (2.3%)	5,286 (2.2%)			
20–24	4,463 (22%)	49,871 (20%)			
25–29	6,452 (32%)	75,574 (31%)			
30–34	5,118 (25%)	64,516 (26%)			
35–39	2,862 (14%)	37,283 (15%)			
40–44	815 (4.0%)	11,636 (4.8%)			
≥45	30 (0.1%)	490 (0.2%)			
**Maternal ethnic group (Bedouin)** ^*^	15,506 (77%)	130,708 (53%)	<0.001	0.5	0.02
**Maternal obesity** ^*^	256 (1.3%)	1,101 (0.5%)	<0.001	0.09	0.01
**Maternal smoking during pregnancy**	98 (0.5%)	933 (0.4%)	0.023	0.02	0
**Maternal diabetes** ^*^	189 (0.9%)	804 (0.3%)	<0.001	0.08	0.02
**Maternal comorbidity indicating NSAID Tx** ^*^	2,717 (13%)	22,863 (9.3%)	<0.001	0.13	0
**Maternal exposure to other antipyretics** ^*^	12,103 (60%)	32,715 (13%)	<0.001	1.1	0
**Gravidity** ^*^	4 (2, 6)	3 (2, 5)	<0.001	0.24	0.02
**Pregnancy age, days**	276 (266, 281)	275 (266, 281)	<0.001	0.05	0.02
Missing	50	661			
**Conception by assisted reproductive technology**	88 (0.4%)	831 (0.3%)	0.026	0.02	0.01
**Conception by insemination**	11 (<0.1%)	174 (<0.1%)	0.4	0.01	0.02
**Conception by IVF**	78 (0.4%)	715 (0.3%)	0.019	0.02	0.01
**Lack of prenatal care** ^*^	193 (1.0%)	1,628 (0.7%)	<0.001	0.03	0.01
**Folic acid** ^*^	9,028 (45%)	34,264 (14%)	<0.001	0.72	0.02
**Sex of newborn (males)**	10,350 (51%)	124,573 (51%)	0.8	0	0
Missing	50	1,578			
**Pregnancy termination**	50 (0.2%)	1,578 (0.6%)	<0.001	0.06	.07

ESS for matching-adjusted model targeting the ATE were 4,910 exposed and 236,721 controls. No actual units were discarded during the proccess.

Maternal obesity was defined using ICD-9 codes 278.0–278.4, diabetes using ICD-9 250.* and gestational diabetes (GDM) 648.00–648.04, and folic acid use as dispensation during the first trimester. Maternal smoking was based on self-report or ICD-9 code 305.1. Exposure to other analgesics or antipyretics was defined as first-trimester exposure to either acetaminophen or dipyrone. NSAID indications were defined as first-trimester diagnoses for conditions commonly treated with NSAID, including musculoskeletal/joint disorders, pain and inflammatory conditions, injuries/fractures, and pregnancy-related diagnoses such as threatened abortion or fever/infection. Gravidity refers to total prior pregnancies.

Missing values for fetal sex reflect pregnancy terminations due to suspected fetal malformations, for which fetal sex could not be determined.

SMDs for *Calendar year of birth* were calculated using the ordinal calendar year variable; grouped categories are shown for presentation purposes only.

^1^*n* (%); Median (Q1, Q3)..

^2^Differences between groups were assessed using Wilcoxon rank sum test for continuous variables and Pearson’s Chi-squared test for categorical variables.

*Covariates included in adjusted models.

aSMD, absolute Standardized Mean Difference; ATE, Average Treatment Effect; ESS, Effective Sample Size; NSAID, Nonsteroidal anti-inflammatory drugs; Tx, Treatment.

### Total NSAID exposure

**Fig 4:**
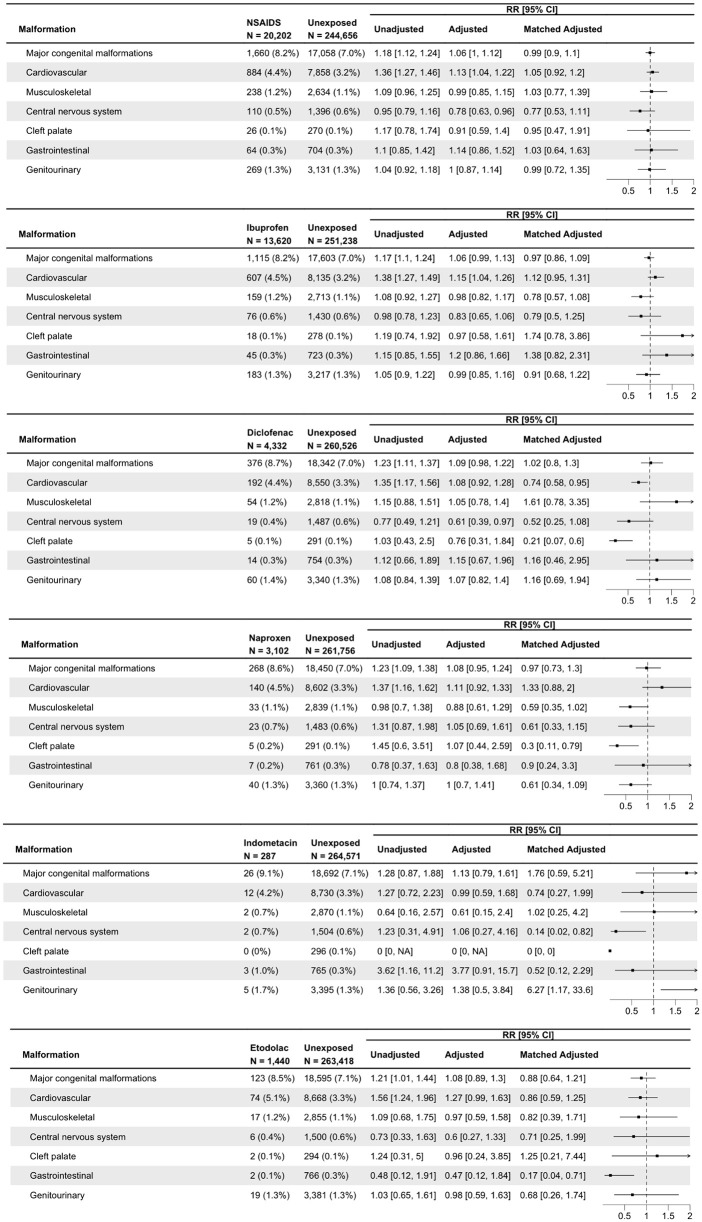
Crude, adjusted, and matched relative risks for total and individual NSAID exposure in relation to major congenital malformations overall and by organ system. NSAID, NonSteroidal Anti-Inflammatory Drugs. The figure presents crude, matched, and optimally full-matched adjusted relative risks (aRRs) with 95% confidence intervals for major congenital malformations overall and by organ system, comparing NSAID-exposed and unexposed pregnancies. The forest plot depicts the matched aRRs, with malformation prevalence shown alongside the regression results. Any NSAID exposure included ibuprofen, diclofenac, naproxen, etodolac, indomethacin, piroxicam, and lornoxicam; analyses by specific agent were presented for ibuprofen, diclofenac, naproxen, etodolac, and indomethacin. Models were matched and adjusted for maternal age, ethnicity, lack of perinatal care, diabetes, obesity, folic acid supplementation, gravidity, calendar year, smoking, NSAID indication, and exposure to other analgesics or antipyretics.

Among 20,202 NSAID-exposed pregnancies, 1,651 (8.2%) were diagnosed with MCMs, compared with 16,998 (7.0%) among the 244,656 unexposed pregnancies. NSAID exposure was associated with an increased risk of MCMs in crude analyses (RR = 1.18 (95% CI[1.12,1.24])); however, no association was found after matching (matched-aRR = 0.99 (95% CI [0.90,1.10])) (Fig 4). Cardiovascular malformations were identified in 885 (4.4%) of exposed and 7,873 (3.2%) of unexposed pregnancies (*p* < 0.001), with a higher crude risk (RR = 1.36 (95% CI [1.27,1.46])) that did not persist in the matched analysis (matched-aRR = 1.05 (95% CI [0.92,1.20])). No evidence of increased risk for musculoskeletal malformations (matched-aRR = 1.03 (95% CI [0.77,1.39])), central nervous system malformations (matched-aRR = 0.77 (95% CI [0.53,1.11])), cleft palate (matched-aRR = 0.95 (95% CI [0.47–1.91])), gastrointestinal malformations (matched-aRR = 1.03 (95% CI [0.64–1.63])), or genitourinary malformations (matched-aRR = 0.99 (95% CI [0.72,1.35])) was observed.

### Specific NSAID exposure

No associations were observed between exposure to any of the NSAID and the risk of overall MCMs (ibuprofen: matched-aRR = 0.97 (95% CI [0.86–1.09]); diclofenac: matched-aRR = 1.02 (95% CI [0.80,1.30]); naproxen: matched-aRR = 0.97 (95% CI [0.73,1.30]); indomethacin: matched-aRR = 1.76 (95% CI [0.59, 5.21]); etodolac: matched-aRR = 0.88 (95% CI [0.64, 1.21])). No significant associations were observed between individual NSAID and congenital malformations across different organ systems (Fig 4).

### Dose–response analysis

The prevalence of MCMs was 8.2% among short-term NSAID users (1–7 DDD), 8.6% among medium-term users (8–21 DDD), and 9.3% among long-term users (>21 DDD), compared with 7.0% in the unexposed group. No association was found with MCMs across the different dosage categories (Table B1 in S1 File).

### Sensitivity analysis

In simulations, the adjusted risk ratio increased as a larger proportion of the cohort was assumed to be misclassified and reassigned from unexposed to exposed. Using the 1.3% missed-exposure estimate from prior validation work at SUMC as a plausible degree of misclassification, reclassifying 1.3% of the cohort [42] (with an 8.3% MCMs prevalence among reclassified individuals) yielded a nonsignificant association in the matching-adjusted analysis (matched-aRR = 1.06 (95% CI [0.97,1.15])). Overall, plausible levels of unrecorded over-the-counter ibuprofen exposure did not materially change the direction or statistical inference of the primary results (Appendix A2 in S1 File).

Concerning the distribution of indications among exposed and unexposed pregnancies, musculoskeletal and pain or inflammatory indications were rare in both groups (<0.1%), while fever or infections and pregnancy-related indications were more common among exposed pregnancies (3.6% versus 2.0% and 9.9% versus 7.0%, respectively) (Table A1 in S1 File). Additional sensitivity analyses, performed to address potential temporal ambiguity and collider bias from concurrent use of other analgesics, also showed no association with overall or organ-specific malformations (Tables A2 and A3 in S1 File). Extending NSAID exposure to two weeks before conception yielded similar results (matched-aRR = 1.02 (95% CI [0.90,1.12])).

## Discussion

In this matched population-based cohort study, including more than 265,000 singleton pregnancies, exposure to NSAID during the first trimester was not associated with increased risk of MCMs, both overall and according to specific organ systems. Consistently, no dose-response was observed between cumulative NSAID exposure and malformation risk.

The consistency of results after exclusion of pregnancies exposed to acetaminophen suggests that our findings are not influenced by concomitant use of alternative analgesics. The use of ibuprofen over the study period showed an inverted U–shape, peaking around 2010. This pattern may reflect temporal changes in prescribing practices or public health guidance.

The study population comprises approximately 55% women of Bedouin origin. The Israeli Bedouins are one of the largest Bedouin communities worldwide, with others living in the Middle East, the Arabian Peninsula, and North Africa. The Bedouin are characterized by distinct demographic and social features, including historically semi‑nomadic roots and relatively high rates of consanguinity compared to other Israeli populations [43]. While these characteristics may differ from those in other settings, the findings contribute important data that complement studies from diverse global populations. The safety of NSAID use in pregnancy has been evaluated in multiple cohorts, with findings both supporting and contrasting our results. Several large studies, including a registry-based analysis of over 91,000 pregnancies by Nezvalová-Henriksen and colleagues [16] and self-reported cohorts such as van Gelder and colleagues [13], found no significant association between first-trimester NSAID exposure and congenital malformations. Likewise, analyses of specific agents such as diclofenac reported no excess risk [19]. Our study extends this evidence by comprehensively evaluating individual NSAID and MCMs across all organ-system categories. Using detailed dispensation data and matched, adjusted analyses with follow-up through the first year of life, we were able to assess both overall and organ-specific risks for each drug, enhancing the detection of potential drug-specific signals while minimizing bias.

Conversely, several studies have reported findings that differ from ours. A cohort of 37,000 pregnancies found an increased risk of cardiovascular malformations associated with NSAID prescriptions [12], while comparative studies between NSAID and acetaminophen reported elevated risks for multiple anomalies but did not adjust for indication bias [17]. A nationwide Korean study including over 100,000 exposed pregnancies also observed higher rates of major and cardiovascular malformations, though these results were limited by reliance on prescription data [44]. Additional reports have described possible drug-specific associations, including ibuprofen with spina bifida, aspirin with anencephaly or encephalocele, and naproxen with encephalocele or oral clefts [9]. Unlike previous studies, ours used dispensation data, covering both prescription and over-the-counter NSAID and included pregnancy terminations, reducing exposure misclassification and bias toward the null. Follow-up through the first year of life further captured malformations diagnosed after birth, offering a more complete risk assessment. Despite these strengths, we found no association between NSAID exposure and congenital malformations.

Our study possesses several strengths. First, this analysis was based on a large, diverse population, encompassing over 265,000 pregnancies across a broad demographic spectrum. This diversity strengthens the external validity and generalizability of the findings across populations and clinical settings. By including pregnancy terminations, the study captured malformations that would otherwise be missed in birth-only cohorts, yielding a more complete and less biased risk assessment [45]. Follow-up through the first year of life further identified malformations diagnosed beyond the neonatal period, improving the completeness and accuracy of case ascertainment [46]. A dose-response analysis assessing cumulative NSAID exposure showed no trend toward increased malformation risk, supporting the robustness of the primary findings. Finally, the matching for a wide range of maternal, demographic, and pregnancy-related characteristics reduced the likelihood of residual confounding. Together, these methodological advantages enhance internal validity and may explain the conflicting results between our study and previous studies [9,12,17,44].

This study has several limitations. First, as in other pharmacoepidemiologic investigations, exposure to NSAID was determined from dispensation records rather than confirmed intake. Although this approach does not capture actual ingestion, prior work has shown high concordance between prescription data and medication use, particularly among pregnant women, and dispensation data have been validated as a reliable source for evaluating drug safety in pregnancy [47–50], including associations with congenital malformations [51]. Second, our dataset did not capture spontaneous abortions. This omission may lead to underestimation of early pregnancy losses potentially related to teratogenic exposures; however, spontaneous abortion is inherently difficult to ascertain, particularly in early gestation when medical care may not be sought. Third, ibuprofen’s over-the-counter availability could lead to minor exposure misclassification. However, nearly all dispensations occur through Clalit or affiliated pharmacies, with only a small proportion of private pharmacies operating independently [52]. In a 2014 validation study, 1.3% of women reported purchases outside the network [53], a proportion likely even smaller in recent years [52]. Sensitivity analyses simulating up to 3% misclassification indicated that any resulting bias was not significant (Appendix A2 in S1 File). Some variables commonly considered potential confounders, such as socioeconomic status and maternal education, were unavailable in our dataset. If these factors are associated with both NSAID exposure and MCMs, their omission could introduce residual confounding. However, by clustering analyses by maternal ID, we partially accounted for maternal characteristics that are consistent across pregnancies, including prior adverse outcomes and other unmeasured factors, potentially reducing bias from unmeasured confounders. Although clinicians may have had access to routine clinical information, including medication history as part of standard care, diagnoses were made prior to and independently of the research question and were not influenced by exposure classification within this study.

In this large, population-based cohort, we found no evidence supporting an association between exposure to NSAID during the first trimester and overall MCMs or specific organ system malformations. We believe these findings have significant clinical relevance given current concerns [1] regarding pain and fever treatment during pregnancy; the comprehensive approach of this study provides a reliable assessment of risk with important implications for both clinical practice and future research in maternal-fetal medicine. While the results are reassuring, further research is needed to provide additional confirmation.

### Ethical approval

The research was approved by the Soroka University Medical Center ethics committee in accordance with the Declaration of Helsinki (study number: 0069–20-SOR, Approval date: 07/03/2022). Due to the retrospective nature of the cohort, the ethic committee waived the need of informed consent to participate.

### Consent to participate

Due to the retrospective nature of the cohort, the ethic committee waived the need of informed consent to participate.

### Consent to publish

Due to the retrospective nature of the cohort, the ethic committee waived the need of informed consent to publish.

## Supporting information

S1 File Supplementary Materials. **Appendix A1:** Dataset Assembly. **Appendix A2:** Potential Misclassification Bias and Sensitivity Analyses. **Table A1:** Distribution of clinical indications for NSAID use among exposed and unexposed pregnancies. **Fig A1:** NSAID and Other Antipyretics exposures and intersections (UpSet plot). **Table A2:** Association of first-trimester NSAID exposure with congenital malformations in sensitivity analyses addressing potential bias related to other antipyretic exposure. **Table A3:** Association of first-trimester NSAID exposure with congenital malformations excluding pregnancies subjected to other antipyretic in the first trimester. **Table B1:** Adjusted associations between total Defined Daily Dose (DDD) of NSAID dispensed during the first trimester and overall risk of major malformations. **Table C1:** Comparison of maternal and pregnancy characteristics between pregnancies exposed and unexposed to Ibuprofen during the first trimester. **Table C2:** Crude, adjusted, and matched relative risks for Ibuprofen exposure in relation to major congenital malformations overall and by specific organ systems. **Table C3:** Adjusted associations between total Defined Daily Dose (DDD) of Ibuprofen dispensed during the first trimester and overall risk of major malformations. **Table C4:** Comparison of maternal and pregnancy characteristics between pregnancies exposed and unexposed to Diclofenac during the first trimester. **Table C5:** Crude, adjusted, and matched relative risks for Diclofenac exposure in relation to major congenital malformations overall and by specific organ systems. **Table C6:** Adjusted associations between total Defined Daily Dose (DDD) of Diclofenac dispensed during the first trimester and overall risk of major malformations. **Table C7:** Comparison of maternal and pregnancy characteristics between pregnancies exposed and unexposed to Naproxen during the first trimester. **Table C8:** Crude, adjusted, and matched relative risks for Naproxen exposure in relation to major congenital malformations overall and by specific organ systems. **Table C9:** Adjusted associations between total Defined Daily Dose (DDD) of Naproxen dispensed during the first trimester and overall risk of major malformations. **Table C10:** Comparison of maternal and pregnancy characteristics between pregnancies exposed and unexposed to Indomethacin during the first trimester. **Table C11:** Crude, adjusted, and matched relative risks for Indomethacin exposure in relation to major congenital malformations overall and by specific organ systems. **Table C12:** Adjusted associations between total Defined Daily Dose (DDD) of Indomethacin dispensed during the first trimester and overall risk of major malformations. **Table C13:** Comparison of maternal and pregnancy characteristics between pregnancies exposed and unexposed to Etodolac during the first trimester. **Table C14:** Crude, adjusted, and matched relative risks for Etodolac exposure in relation to major congenital malformations overall and by specific organ systems. **Table C15:** Adjusted associations between total Defined Daily Dose (DDD) of Etodolac dispensed during the first trimester and overall risk of major malformations. **Table C16:** Comparison of maternal and pregnancy characteristics between pregnancies exposed and unexposed to Piroxicam during the first trimester. **Table C17:** Crude, adjusted, and matched relative risks for Piroxicam exposure in relation to major congenital malformations overall and by specific organ systems. **Table C18:** Adjusted associations between total Defined Daily Dose (DDD) of Piroxicam dispensed during the first trimester and overall risk of major malformations. **Table C19:** Comparison of maternal and pregnancy characteristics between pregnancies exposed and unexposed to Lornoxicam during the first trimester. **Table C20:** Crude, adjusted, and matched relative risks for Lornoxicam exposure in relation to major congenital malformations overall and by specific organ systems. **Table C21:** Adjusted associations between total Defined Daily Dose (DDD) of Lornoxicam dispensed during the first trimester and overall risk of major malformations. **STROBE Checklist D1:** checklist of items that should be included in reports of observational studies. *von Elm E, Altman DG, Egger M, Pocock SJ, Gøtzsche PC, et al. (2007) The Strengthening the Reporting of Observational Studies in Epidemiology (STROBE) Statement: Guidelines for Reporting Observational Studies. PLOS Medicine 4(10): e296.* https://doi.org/10.1371/journal.pmed.0040296.
(DOCX)

## Abbreviations

aRR: adjusted-Relative Risk
CHS: Clalit Health Services
DDD: defined-daily-dose
GDM: gestational diabetes mellitus
ICD-9: International Classification of Diseases, 9th Revision
IQR: Interquartile Range
MACDP: Metropolitan Atlanta Congenital Defects Program
MCMs: major congenital malformations
NSAID: nonsteroidal anti-inflammatory drugs
siPREG: Southern Israeli Pregnancy Registry
