# Supplementary material for: First-trimester nonsteroidal anti-inflammatory drugs exposure and risk of major congenital malformations: A retrospective register-based cohort study
Source: PLoS Med. 2026 May 14;23(5):e1005063. doi: 10.1371/journal.pmed.1005063 (PMC13175353; doi:10.1371/journal.pmed.1005063)
Supplement: S1 File — Appendix A1: Dataset Assembly. Appendix A2: Potential Misclassification Bias and Sensitivity Analyses. Table A1: Distribution of clinical indications for NSAID use among exposed and unexposed pregnancies. Fig A1: NSAID and Other Antipyretics exposures and intersections (UpSet plot). Table A2: Association of first-trimester NSAID exposure with congenital malformations in sensitivity analyses addressing potential bias related to other antipyretic exposure. Table A3: Association of first-trimester NSAID exposure with congenital malformations excluding pregnancies subjected to other antipyretic in the first trimester. Table B1: Adjusted associations between total Defined Daily Dose (DDD) of NSAID dispensed during the first trimester and overall risk of major malformations. Table C1: Comparison of maternal and pregnancy characteristics between pregnancies exposed and unexposed to Ibuprofen during the first trimester. Table C2: Crude, adjusted, and matched relative risks for Ibuprofen exposure in relation to major congenital malformations overall and by specific organ systems. Table C3: Adjusted associations between total Defined Daily Dose (DDD) of Ibuprofen dispensed during the first trimester and overall risk of major malformations. Table C4: Comparison of maternal and pregnancy characteristics between pregnancies exposed and unexposed to Diclofenac during the first trimester. Table C5: Crude, adjusted, and matched relative risks for Diclofenac exposure in relation to major congenital malformations overall and by specific organ systems. Table C6: Adjusted associations between total Defined Daily Dose (DDD) of Diclofenac dispensed during the first trimester and overall risk of major malformations. Table C7: Comparison of maternal and pregnancy characteristics between pregnancies exposed and unexposed to Naproxen during the first trimester. Table C8: Crude, adjusted, and matched relative risks for Naproxen exposure in relation to major congenital malformations overall an [file pmed.1005063.s001.docx]

Supplementary Materials
First Trimester Nonsteroidal Anti-inflammatory Drugs Exposure and Risk of Major Congenital Malformations: a retrospective register-based cohort study

Ariel Avraham Hasidim MD, MPH, Itamar Ben Shitrit* MD, MPH, Daphna Idan, MD, MPH, [Tal Michael](https://protect.checkpoint.com/v2/___https://bpspubs.onlinelibrary.wiley.com/authored-by/Michael/Tal___.YzJlOmJlbmd1cmlvbnVuaXZlcnNpdHlvZnRoZW5lZ2V2OmM6bzpjYTZlMjk2MmQ2NGFhMDQyMmIxZjI3ZGQzMGQ1MTg3MTo2OjkwMmE6M2VkNjRhNWVlNGM0NjE1NmU0ZDJiOWJkMWEwMDE4MTgzMTE5ZTI0OTVmNmZjNjYwMmVmZmM1MjY4M2QxZDI0MTpwOlQ6Tg) MD, MPH, PhD, Amalia Levy MPH, PhD , [Gali Pariente](https://protect.checkpoint.com/v2/___https://bpspubs.onlinelibrary.wiley.com/authored-by/Pariente/Gali___.YzJlOmJlbmd1cmlvbnVuaXZlcnNpdHlvZnRoZW5lZ2V2OmM6bzpjYTZlMjk2MmQ2NGFhMDQyMmIxZjI3ZGQzMGQ1MTg3MTo2Ojk1NDA6N2U0NDNmYjM2YTFlYzdjMWU1ZGI5NGY3ZDA2YmI2OGQzZjg0YzFlNzc3MDVjZWYyZTQ2NjgzYmI5ZDBiMmQxMDpwOlQ6Tg) MD, Eitan Lunenfeld MD, MHA, Sharon Daniel MD, MPH, PhD

[Chapter 1 – Supplemental Methods and Sensitivity and Bias Analyses 3](#_Toc227041936)

[Appendix A1: Dataset Assembly 3](#_Toc227041937)

[Appendix A2: Potential Misclassification Bias and Sensitivity Analyses: 3](#_Toc227041938)

[Table A1: Distribution of clinical indications for NSAID use among exposed and unexposed pregnancies 5](#_Toc227041939)

[Figure A1: NSAIDs and Other Antipyretics exposures and intersections (UpSet plot) 6](#_Toc227041940)

[Table A2: Association of first-trimester NSAID exposure with congenital malformations in sensitivity analyses addressing potential bias related to other antipyretic exposure 7](#_Toc227041941)

[Table A3: Association of first-trimester NSAID exposure with congenital malformations excluding pregnancies subjected to other antipyretic in the first trimester 8](#_Toc227041942)

[Chapter 2 – Primary Analysis 9](#_Toc227041943)

[**Table B1** – Adjusted associations between total Defined Daily Dose (DDD) of NSAIDs dispensed during the first trimester and overall risk of major malformations 9](#_Toc227041944)

[Chapter 3 – Secondary Analysis 10](#_Toc227041945)

[Ibuprofen 10](#_Toc227041946)

[Table C1 – Comparison of maternal and pregnancy characteristics between pregnancies exposed and unexposed to Ibuprofen during the first trimester. 10](#_Toc227041947)

[Table C2 – Crude, adjusted, and matched relative risks for Ibuprofen exposure in relation to major congenital malformations overall and by specific organ systems 12](#_Toc227041948)

[Table C3 – Adjusted associations between total Defined Daily Dose (DDD) of Ibuprofen dispensed during the first trimester and overall risk of major malformations 13](#_Toc227041949)

[Diclofenac 14](#_Toc227041950)

[Table C4 – Comparison of maternal and pregnancy characteristics between pregnancies exposed and unexposed to Diclofenac during the first trimester. 14](#_Toc227041951)

[Table C5 – Crude, adjusted, and matched relative risks for Diclofenac exposure in relation to major congenital malformations overall and by specific organ systems 16](#_Toc227041952)

[Table C6 – Adjusted associations between total Defined Daily Dose (DDD) of Diclofenac dispensed during the first trimester and overall risk of major malformations 17](#_Toc227041953)

[Naproxen 18](#_Toc227041954)

[Table C7 – Comparison of maternal and pregnancy characteristics between pregnancies exposed and unexposed to Naproxen during the first trimester. 18](#_Toc227041955)

[Table C8 – Crude, adjusted, and matched relative risks for Naproxen exposure in relation to major congenital malformations overall and by specific organ systems 20](#_Toc227041956)

[Table C9 – Adjusted associations between total Defined Daily Dose (DDD) of Naproxen dispensed during the first trimester and overall risk of major malformations 21](#_Toc227041957)

[Indomethacin 22](#_Toc227041958)

[Table C10 – Comparison of maternal and pregnancy characteristics between pregnancies exposed and unexposed to Indomethacin during the first trimester. 22](#_Toc227041959)

[Table C11 – Crude, adjusted, and matched relative risks for Indomethacin exposure in relation to major congenital malformations overall and by specific organ systems 24](#_Toc227041960)

[Table C12 – Adjusted associations between total Defined Daily Dose (DDD) of Indomethacin dispensed during the first trimester and overall risk of major malformations 25](#_Toc227041961)

[Etodolac 26](#_Toc227041962)

[Table C13 – Comparison of maternal and pregnancy characteristics between pregnancies exposed and unexposed to Etodolac during the first trimester. 26](#_Toc227041963)

[Table C14 – Crude, adjusted, and matched relative risks for Etodolac exposure in relation to major congenital malformations overall and by specific organ systems 28](#_Toc227041964)

[Table C15 – Adjusted associations between total Defined Daily Dose (DDD) of Etodolac dispensed during the first trimester and overall risk of major malformations 29](#_Toc227041965)

[Piroxicam 30](#_Toc227041966)

[Table C16 – Comparison of maternal and pregnancy characteristics between pregnancies exposed and unexposed to Piroxicam during the first trimester. 30](#_Toc227041967)

[Table C17 – Crude, adjusted, and matched relative risks for Piroxicam exposure in relation to major congenital malformations overall and by specific organ systems 32](#_Toc227041968)

[Table C18 – Adjusted associations between total Defined Daily Dose (DDD) of Piroxicam dispensed during the first trimester and overall risk of major malformations 33](#_Toc227041969)

[Lornoxicam 34](#_Toc227041970)

[Table C19 – Comparison of maternal and pregnancy characteristics between pregnancies exposed and unexposed to Lornoxicam during the first trimester. 34](#_Toc227041971)

[Table C20 – Crude, adjusted, and matched relative risks for Lornoxicam exposure in relation to major congenital malformations overall and by specific organ systems 36](#_Toc227041972)

[Table C21 – Adjusted associations between total Defined Daily Dose (DDD) of Lornoxicam dispensed during the first trimester and overall risk of major malformations 37](#_Toc227041973)

[Chapter 4 – STROBE checklist 38](#_Toc227041974)

[Appendix Checklist D1: STROBE Statement: checklist of items that should be included in reports of observational studies 38](#_Toc227041975)

# Chapter 1 – Supplemental Methods and Sensitivity and Bias Analyses

## Appendix A1: Dataset Assembly

The cohort was established by merging four different databases. Data on pregnancy and delivery outcomes were obtained from the Obstetrics and Gynecology Division database at SUMC, which includes demographic characteristics, pregnancy-related diagnoses, and delivery outcomes. Data concerning major congenital malformations were retrieved from SUMC's hospitalization database, which documents malformations diagnosed up to the age of one year. Data on malformations diagnosed prior to elective pregnancy terminations, performed due to suspected fetal malformation, were manually extracted from the registry of the Committee for Termination of Pregnancies at SUMC. These data encompassed maternal and pregnancy characteristics, as well as major fetal malformations identified through ultrasound or echocardiography. All malformation diagnoses were assigned by board-certified neonatologists or pediatricians and were coded according to the International Classification of Diseases, 9th Revision (ICD-9). Medication dispensation data were obtained from the CHS database, which documents all dispensations of prescription and over-the-counter medications nationwide, including drug names, Anatomical Therapeutic Chemical (ATC) classification codes, and the dosage dispensed in defined daily doses (DDD). The databases were merged using each individual's unique national identification number and the hospitalization number for each SUMC admission, allowing linkage of maternal records with corresponding newborn or termination data.

## Appendix A2: Potential Misclassification Bias and Sensitivity Analyses:

A potential limitation of our study is the availability of ibuprofen as an over-the-counter medication. Most sales in Israel occur through health service clinics or pharmacies affiliated with Clalit Health Services, and dispensations from these sources are captured in our data (with or without prescription). However, a small number of independent, non-affiliated pharmacies are not recorded and may result in exposure misclassification, whereby some truly exposed individuals are classified as unexposed. While the classic expectation is that non-differential exposure misclassification attenuates associations toward the null, we specifically consider the less typical—but more consequential—scenario in which this misclassification could bias the estimated association away from the null.

To quantify the potential impact of unrecorded over-the-counter ibuprofen use, we conducted a bootstrap-based probabilistic sensitivity (tipping-point) analysis in which an increasing proportion of individuals were randomly reclassified from the unexposed to the exposed group. Because dispensation records are expected to be highly specific but potentially insensitive, we treated recorded exposure as a reliable marker and assumed that the prevalence of major malformations among the recorded exposed group reflects the true prevalence among potentially misclassified exposed individuals. Accordingly, at each step we randomly selected individuals from the unexposed group such that 8.3% had major malformations and reassigned them to the exposed group. The reassigned proportion ranged from 0% to 3% of the total cohort in 0.05% increments, with 100 random reallocations per step.

In a prior validation study conducted in the same source population[1], we estimated that reliance on dispensation data could miss approximately 1.3% of true ibuprofen exposures. Consistent with this estimate, our tipping-point analysis indicates that reclassifying 1.3% of the cohort from unexposed to exposed (assuming a major malformation prevalence of 8.3% among the reclassified individuals) yields a non-significant association in the matching-adjusted model (aRR = 1.06 (95%CI [0.97,1.15])), suggesting that plausible levels of exposure misclassification are unlikely to materially alter our conclusions.

*1. Daniel S, Koren G, Lunenfeld E, Bilenko N, Ratzon R, Levy A. Fetal exposure to nonsteroidal anti-inflammatory drugs and spontaneous abortions. CMAJ [Internet]. 2014 Mar 18 [cited 2025 Oct 8];186(5). Available from: https://pubmed.ncbi.nlm.nih.gov/24491470/*

## Table A1: Distribution of clinical indications for NSAID use among exposed and unexposed pregnancies

| **Characteristic** | **NSAIDS** N = 20,212*^1^* | **Unexposed** N = 244,931*^1^* | **p-value***^2^* |
| --- | --- | --- | --- |
| **Musculoskeletal** | 7 (<0.1%) | 2 (<0.1%) | <0.001 |
| **Pain / Inflammatory** | 17 (<0.1%) | 110 (<0.1%) | 0.014 |
| **Injury / Fracture** | 429 (2.1%) | 3,612 (1.5%) | <0.001 |
| **Pregnancy-Related** | 2,008 (9.9%) | 17,242 (7.0%) | <0.001 |
| **Fever / Infection** | 724 (3.6%) | 4,940 (2.0%) | <0.001 |
| *^1^*n (%) | | | |
| *^2^*Fisher's exact test; Pearson's Chi-squared test | | | |

##

##

## Figure A1: NSAIDs and Other Antipyretics exposures and intersections (UpSet plot)


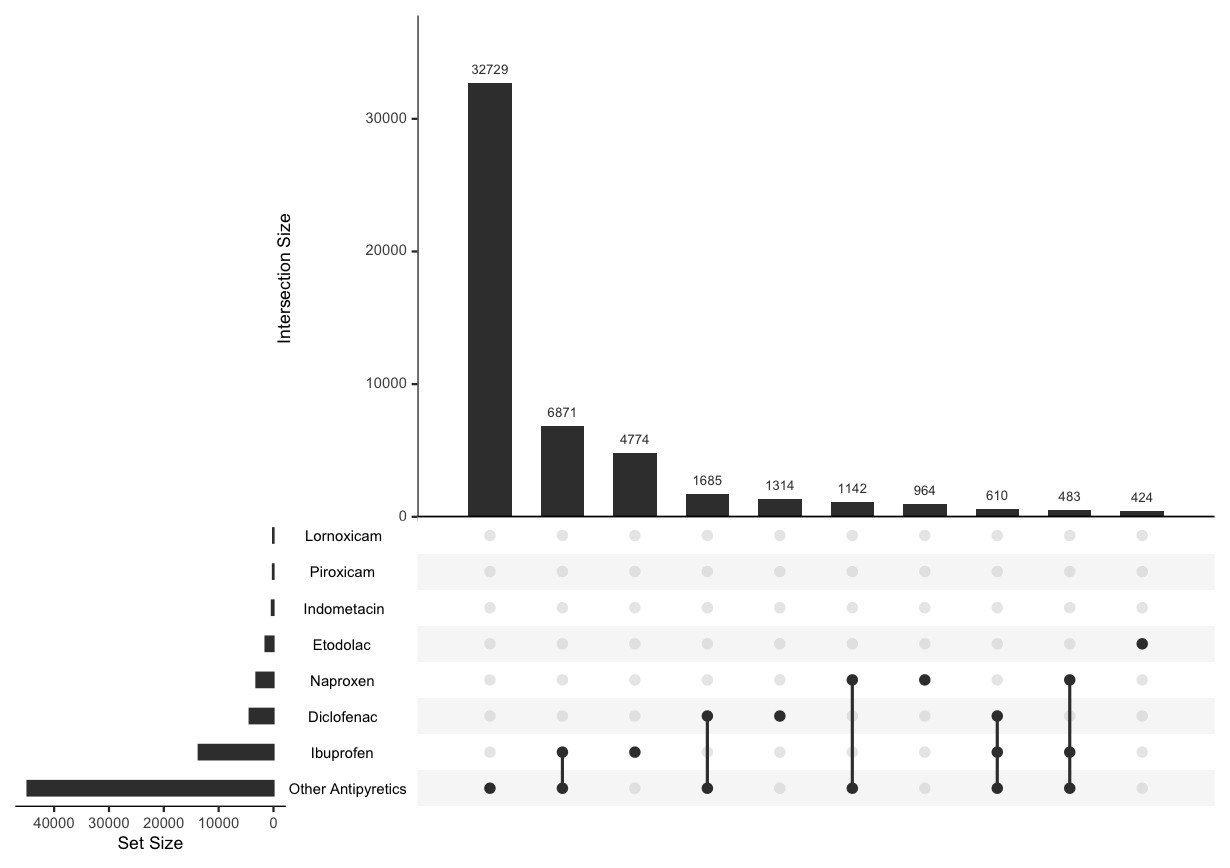


The UpSet technique visualizes set intersections in a matrix layout and introduces aggregates based on grouping. The data underlying this figure are available as CSV files at: https://github.com/arielhasidim/nsaids-major-malformations-2026.

## Table A2: Association of first-trimester NSAID exposure with congenital malformations in sensitivity analyses addressing potential bias related to other antipyretic exposure

|  |  |  | **RR** [95% CI] | | |
| --- | --- | --- | --- | --- | --- |
|  | **NSAID** (N=20202) | **Unexposed** (N=244656) | Model 1*^1^* | Model 2*^2^* | Model 3*^2^* |
| Major congenital malformations | 1,660 (8.2%) | 17,058 (7.0%) | 1.01 [0.93, 1.1] | 1.02 [0.94, 1.11] | 1 [0.92, 1.09] |
| Cardiovascular | 884 (4.4%) | 7,858 (3.2%) | 1.11 [0.99, 1.24] | 1.11 [0.99, 1.25] | 1.1 [0.98, 1.24] |
| Musculoskeletal | 238 (1.2%) | 2,634 (1.1%) | 1 [0.8, 1.24] | 1.03 [0.83, 1.26] | 1.02 [0.82, 1.27] |
| Central nervous system | 110 (0.5%) | 1,396 (0.6%) | 0.82 [0.59, 1.14] | 0.75 [0.54, 1.03] | 0.72 [0.51, 1.02] |
| Cleft palate | 26 (0.1%) | 270 (0.1%) | 0.83 [0.5, 1.38] | 0.75 [0.46, 1.23] | 0.81 [0.49, 1.32] |
| Gastrointestinal | 64 (0.3%) | 704 (0.3%) | 0.99 [0.65, 1.5] | 1.03 [0.7, 1.51] | 0.95 [0.63, 1.43] |
| Genitourinary | 269 (1.3%) | 3,131 (1.3%) | 0.9 [0.74, 1.11] | 0.93 [0.77, 1.12] | 0.9 [0.74, 1.1] |
| *^1^*ESS for matching-adjusted model targeting the ATE were 7,696 exposed and 240,136 controls. No actual units were discarded during the proccess. | | | | | |
| *^2^*ESS for matching-adjusted model targeting the ATE were 7,288 exposed and 239,879 controls. No actual units were discarded during the proccess. | | | | | |
| ATE, Average Treatment Effect; ESS, Effective Sample Size; NSAIDS, Nonsteroidal anti-inflammatory drugs. | | | | | |
| **Model 1** was matched and adjusted for birth termination year and its quadratic term, maternal age, pregnancy number, lack of prenatal care, Bedouin population group, NSAID indication, and maternal diabetes, obesity, folic acid use, and smoking. **Model 2** included all Model 1 covariates, with additional matching and adjustment for exposure to other antipyretics before NSAID exposure. **Model 3** used the same design as Model 2, with additional adjustment, but not matching, for exposure to other antipyretics after NSAID exposure. | | | | | |

##

## Table A3: Association of first-trimester NSAID exposure with congenital malformations excluding pregnancies subjected to other antipyretic in the first trimester

|  | **NSAID** (N=8099) | **Unexposed** (N=211941) | **RR** [95% CI] |
| --- | --- | --- | --- |
| Major congenital malformations | 593 (7.3%) | 14,603 (6.9%) | 0.91 [0.8, 1.03] |
| Cardiovascular | 324 (4.0%) | 6,620 (3.1%) | 1.04 [0.89, 1.21] |
| Musculoskeletal | 82 (1.0%) | 2,261 (1.1%) | 0.74 [0.53, 1.01] |
| Central nervous system | 37 (0.5%) | 1,171 (0.6%) | 0.62 [0.37, 1.07] |
| Cleft palate | 9 (0.1%) | 222 (0.1%) | 0.78 [0.34, 1.77] |
| Gastrointestinal | 25 (0.3%) | 626 (0.3%) | 0.89 [0.54, 1.47] |
| Genitourinary | 94 (1.2%) | 2,712 (1.3%) | 0.83 [0.61, 1.12] |
| Effective sample size for matching-adjusted model targeting the average treatment effect were 3,374 exposed and 211,084 controls. No actual units were discarded during the process. | | | |
| The model was matched and adjusted for birth termination year and its quadratic term, maternal age, pregnancy number, lack of prenatal care, Bedouin population group, NSAID indication, and maternal diabetes, obesity, folic acid use, and smoking, only after the exclusion of all pregnancies exposed to other antipyretics. | | | |

# Chapter 2 – Primary Analysis

### **Table B1** – Adjusted associations between total Defined Daily Dose (DDD) of NSAIDs dispensed during the first trimester and overall risk of major malformations

| Total DDD for NSAIDs | Major Congenital Malformations | Adjusted RR (%95 CI) |
| --- | --- | --- |
| None | 17,654/251,345 (7.0%) | Reference |
| 1-7 | 662/8,022 (8.3%) | 1.06 [0.97, 1.15] |
| 8-21 | 453/5,226 (8.7%) | 1.1 [0.99, 1.22] |
| >21 | 53/550 (9.6%) | 1.24 [0.94, 1.63] |

Models were adjusted for maternal age, ethnicity, lack of perinatal care, diabetes, obesity, folic acid supplementation, gravidity, calendar year, smoking, NSAID indication, and exposure to other analgesics or antipyretics.

# Chapter 3 – Secondary Analysis

## Ibuprofen

### Table C1 – Comparison of maternal and pregnancy characteristics between pregnancies exposed and unexposed to Ibuprofen during the first trimester.

| **Characteristic** | **Ibuprofen** N = 13,620*^1^* | **Unexposed** N = 251,238*^1^* | **p-value***^2^* | **aSMD before** | **aSMD after** |
| --- | --- | --- | --- | --- | --- |
| **Calendar year of birth*^*^*** |  |  | <0.001 | 0.14 | 0.03 |
| 1998–2002 | 1,992 (15%) | 57,330 (23%) |  |  |  |
| 2003–2007 | 3,429 (25%) | 58,563 (23%) |  |  |  |
| 2008–2012 | 4,286 (31%) | 62,906 (25%) |  |  |  |
| 2013–2017 | 3,913 (29%) | 72,439 (29%) |  |  |  |
| **Maternal delivery age, years*^*^*** |  |  | <0.001 | 0.1 | 0.01 |
| <20 | 308 (2.3%) | 5,440 (2.2%) |  |  |  |
| 20–24 | 3,103 (23%) | 51,231 (20%) |  |  |  |
| 25–29 | 4,433 (33%) | 77,593 (31%) |  |  |  |
| 30–34 | 3,427 (25%) | 66,207 (26%) |  |  |  |
| 35–39 | 1,823 (13%) | 38,322 (15%) |  |  |  |
| 40–44 | 514 (3.8%) | 11,937 (4.8%) |  |  |  |
| ≥45 | 12 (<0.1%) | 508 (0.2%) |  |  |  |
| **Maternal ethnic group (Bedouin)*^*^*** | 10,007 (73%) | 136,207 (54%) | <0.001 | 0.41 | 0.05 |
| **Maternal obesity*^*^*** | 160 (1.2%) | 1,197 (0.5%) | <0.001 | 0.08 | 0.01 |
| **Maternal smoking during pregnancy*^*^*** | 52 (0.4%) | 979 (0.4%) | 0.9 | 0 | 0 |
| **Maternal diabetes*^*^*** | 105 (0.8%) | 888 (0.4%) | <0.001 | 0.06 | 0.01 |
| **Maternal comorbidity indicating NSAIDS Tx*^*^*** | 1,730 (13%) | 23,850 (9.5%) | <0.001 | 0.1 | 0.03 |
| **Maternal exposure to other antipyretics** | 8,438 (62%) | 36,380 (14%) | <0.001 | 1.12 | 0 |
| **Gravidity*^*^*** | 4 (2, 6) | 3 (2, 5) | <0.001 | 0.17 | 0.01 |
| **Pregnancy age, days** | 275 (266, 281) | 275 (266, 281) | 0.070 | 0.04 | 0.04 |
| Missing | 28 | 683 |  |  |  |
| **Conception by assisted reproductive technology** | 59 (0.4%) | 860 (0.3%) | 0.079 | 0.01 | 0.01 |
| **Conception by insemination** | 8 (<0.1%) | 177 (<0.1%) | 0.6 | 0 | 0.01 |
| **Conception by IVF** | 52 (0.4%) | 741 (0.3%) | 0.071 | 0.01 | 0 |
| **Lack of prenatal care*^*^*** | 119 (0.9%) | 1,702 (0.7%) | 0.007 | 0.02 | 0.01 |
| **Folic acid*^*^*** | 6,320 (46%) | 36,972 (15%) | <0.001 | 0.73 | 0.01 |
| **Sex of newborn (males)** | 6,974 (51%) | 127,949 (51%) | 0.9 | 0 | 0 |
| Missing | 34 | 1,594 |  |  |  |
| **Pregnancy termination** | 34 (0.2%) | 1,594 (0.6%) | <0.001 | 0.06 | 0.07 |
| *^1^*n (%); Median (Q1, Q3) | | | | | |
| *^2^*Differences between groups were assessed using Wilcoxon rank sum test for continuous variables and Pearson's Chi-squared test for categorical variables. | | | | | |
| *^*^*Covariates included in adjusted models | | | | | |
| aSMD, absolute Standardized Mean Difference; ATE, Average Treatment Effect; ESS, Effective Sample Size; NSAIDS, Nonsteroidal anti-inflammatory drugs; Tx, Treatment. | | | | | |
| ESS for matching-adjusted model targeting the ATE were 3,157 exposed and 247,739 controls. No actual units were discarded during the proccess. | | | | | |
| Maternal obesity was defined using ICD-9 codes 278.0–278.4, diabetes using ICD-9 250.* and gestational diabetes (GDM) 648.00–648.04, and folic acid use as dispensation during the first trimester. Maternal smoking was based on self-report or ICD-9 code 305.1. Exposure to other analgesics or antipyretics was defined as first-trimester exposure to either acetaminophen or dipyrone. NSAID indications were defined as first-trimester diagnoses for conditions commonly treated with NSAIDs, including musculoskeletal/joint disorders, pain and inflammatory conditions, injuries/fractures, and pregnancy-related diagnoses such as threatened abortion or fever/infection. Gravidity refers to total prior pregnancies. | | | | | |
| Missing values for fetal sex reflect pregnancy terminations due to suspected fetal malformations, for which fetal sex could not be determined. | | | | | |
| SMDs for *Calendar year of birth* were calculated using the ordinal calendar year variable; grouped categories are shown for presentation purposes only. | | | | | |

### Table C2 – Crude, adjusted, and matched relative risks for Ibuprofen exposure in relation to major congenital malformations overall and by specific organ systems

| Malformation | Ibuprofen N = 13,620 | Unexposed N = 251,238 | Unadjusted | Adjusted | Matched Adjusted |
| --- | --- | --- | --- | --- | --- |
| Major congenital malformations | 1,115 (8.2%) | 17,603 (7.0%) | 1.17 [1.1, 1.24] | 1.06 [0.99, 1.13] | 0.97 [0.86, 1.09] |
| Cardiovascular | 607 (4.5%) | 8,135 (3.2%) | 1.38 [1.27, 1.49] | 1.15 [1.04, 1.26] | 1.12 [0.95, 1.31] |
| Musculoskeletal | 159 (1.2%) | 2,713 (1.1%) | 1.08 [0.92, 1.27] | 0.98 [0.82, 1.17] | 0.78 [0.57, 1.08] |
| Central nervous system | 76 (0.6%) | 1,430 (0.6%) | 0.98 [0.78, 1.23] | 0.83 [0.65, 1.06] | 0.79 [0.5, 1.25] |
| Cleft palate | 18 (0.1%) | 278 (0.1%) | 1.19 [0.74, 1.92] | 0.97 [0.58, 1.61] | 1.74 [0.78, 3.86] |
| Gastrointestinal | 45 (0.3%) | 723 (0.3%) | 1.15 [0.85, 1.55] | 1.2 [0.86, 1.66] | 1.38 [0.82, 2.31] |
| Genitourinary | 183 (1.3%) | 3,217 (1.3%) | 1.05 [0.9, 1.22] | 0.99 [0.85, 1.16] | 0.91 [0.68, 1.22] |

Models were matched and adjusted for maternal age, ethnicity, lack of perinatal care, diabetes, obesity, folic acid supplementation, gravidity, calendar year, smoking, NSAID indication, and exposure to other analgesics or antipyretics.

### Table C3 – Adjusted associations between total Defined Daily Dose (DDD) of Ibuprofen dispensed during the first trimester and overall risk of major malformations

| Total DDD for Ibuprofen | Major Congenital Malformations | Adjusted RR (%95 CI) |
| --- | --- | --- |
| None | 18,199/257,823 (7.1%) | Reference |
| 1-7 | 541/6,529 (8.3%) | 1.05 [0.96, 1.15] |
| 8-21 | 78/748 (10.4%) | 1.31 [1.04, 1.63] |
| >21 | 4/43 (9.3%) | 1.18 [0.44, 3.14] |

Models were adjusted for maternal age, ethnicity, lack of perinatal care, diabetes, obesity, folic acid supplementation, gravidity, calendar year, smoking, NSAID indication, and exposure to other analgesics or antipyretics.

## Diclofenac

### Table C4 – Comparison of maternal and pregnancy characteristics between pregnancies exposed and unexposed to Diclofenac during the first trimester.

| **Characteristic** | **Diclofenac** N = 4,332*^1^* | **Unexposed** N = 260,526*^1^* | **p-value***^2^* | **aSMD before** | **aSMD after** |
| --- | --- | --- | --- | --- | --- |
| **Calendar year of birth*^*^*** |  |  | <0.001 | 0.14 | 0.05 |
| 1998–2002 | 988 (23%) | 58,334 (22%) |  |  |  |
| 2003–2007 | 1,250 (29%) | 60,742 (23%) |  |  |  |
| 2008–2012 | 1,179 (27%) | 66,013 (25%) |  |  |  |
| 2013–2017 | 915 (21%) | 75,437 (29%) |  |  |  |
| **Maternal delivery age, years*^*^*** |  |  | 0.5 | 0.03 | 0.06 |
| <20 | 95 (2.2%) | 5,653 (2.2%) |  |  |  |
| 20–24 | 881 (20%) | 53,453 (21%) |  |  |  |
| 25–29 | 1,304 (30%) | 80,722 (31%) |  |  |  |
| 30–34 | 1,123 (26%) | 68,511 (26%) |  |  |  |
| 35–39 | 704 (16%) | 39,441 (15%) |  |  |  |
| 40–44 | 215 (5.0%) | 12,236 (4.7%) |  |  |  |
| ≥45 | 10 (0.2%) | 510 (0.2%) |  |  |  |
| **Maternal ethnic group (Bedouin)*^*^*** | 3,772 (87%) | 142,442 (55%) | <0.001 | 0.76 | 0.08 |
| **Maternal obesity*^*^*** | 48 (1.1%) | 1,309 (0.5%) | <0.001 | 0.07 | 0.02 |
| **Maternal smoking during pregnancy*^*^*** | 18 (0.4%) | 1,013 (0.4%) | 0.8 | 0 | 0.05 |
| **Maternal diabetes*^*^*** | 50 (1.2%) | 943 (0.4%) | <0.001 | 0.09 | 0 |
| **Maternal comorbidity indicating NSAIDS Tx*^*^*** | 649 (15%) | 24,931 (9.6%) | <0.001 | 0.17 | 0.03 |
| **Maternal exposure to other antipyretics** | 2,768 (64%) | 42,050 (16%) | <0.001 | 1.12 | 0.01 |
| **Gravidity*^*^*** | 5 (3, 7) | 3 (2, 5) | <0.001 | 0.43 | 0.04 |
| **Pregnancy age, days** | 278 (268, 283) | 275 (266, 281) | <0.001 | 0.07 | 0.01 |
| Missing | 12 | 699 |  |  |  |
| **Conception by assisted reproductive technology** | 12 (0.3%) | 907 (0.3%) | 0.4 | 0.01 | 0.04 |
| **Conception by insemination** | 0 (0%) | 185 (<0.1%) | 0.081 | 0.04 | 0.04 |
| **Conception by IVF** | 12 (0.3%) | 781 (0.3%) | 0.8 | 0 | 0.04 |
| **Lack of prenatal care*^*^*** | 55 (1.3%) | 1,766 (0.7%) | <0.001 | 0.06 | 0.01 |
| **Folic acid*^*^*** | 1,935 (45%) | 41,357 (16%) | <0.001 | 0.66 | 0.08 |
| **Sex of newborn (males)** | 2,186 (51%) | 132,737 (51%) | 0.4 | 0.01 | 0 |
| Missing | 13 | 1,615 |  |  |  |
| **Pregnancy termination** | 13 (0.3%) | 1,615 (0.6%) | 0.008 | 0.05 | 0.03 |
| *^1^*n (%); Median (Q1, Q3) | | | | | |
| *^2^*Differences between groups were assessed using Wilcoxon rank sum test for continuous variables and Pearson's Chi-squared test for categorical variables. | | | | | |
| *^*^*Covariates included in adjusted models | | | | | |
| aSMD, absolute Standardized Mean Difference; ATE, Average Treatment Effect; ESS, Effective Sample Size; NSAIDS, Nonsteroidal anti-inflammatory drugs; Tx, Treatment. | | | | | |
| ESS for matching-adjusted model targeting the ATE were 716 exposed and 260,146 controls. No actual units were discarded during the proccess. | | | | | |
| Maternal obesity was defined using ICD-9 codes 278.0–278.4, diabetes using ICD-9 250.* and gestational diabetes (GDM) 648.00–648.04, and folic acid use as dispensation during the first trimester. Maternal smoking was based on self-report or ICD-9 code 305.1. Exposure to other analgesics or antipyretics was defined as first-trimester exposure to either acetaminophen or dipyrone. NSAID indications were defined as first-trimester diagnoses for conditions commonly treated with NSAIDs, including musculoskeletal/joint disorders, pain and inflammatory conditions, injuries/fractures, and pregnancy-related diagnoses such as threatened abortion or fever/infection. Gravidity refers to total prior pregnancies. | | | | | |
| Missing values for fetal sex reflect pregnancy terminations due to suspected fetal malformations, for which fetal sex could not be determined. | | | | | |
| SMDs for *Calendar year of birth* were calculated using the ordinal calendar year variable; grouped categories are shown for presentation purposes only. | | | | | |

### Table C5 – Crude, adjusted, and matched relative risks for Diclofenac exposure in relation to major congenital malformations overall and by specific organ systems

| Malformation | Diclofenac N = 4,332 | Unexposed N = 260,526 | Unadjusted | Adjusted | Matched Adjusted |
| --- | --- | --- | --- | --- | --- |
| Major congenital malformations | 376 (8.7%) | 18,342 (7.0%) | 1.23 [1.11, 1.37] | 1.09 [0.98, 1.22] | 1.02 [0.8, 1.3] |
| Cardiovascular | 192 (4.4%) | 8,550 (3.3%) | 1.35 [1.17, 1.56] | 1.08 [0.92, 1.28] | 0.74 [0.58, 0.95] |
| Musculoskeletal | 54 (1.2%) | 2,818 (1.1%) | 1.15 [0.88, 1.51] | 1.05 [0.78, 1.4] | 1.61 [0.78, 3.35] |
| Central nervous system | 19 (0.4%) | 1,487 (0.6%) | 0.77 [0.49, 1.21] | 0.61 [0.39, 0.97] | 0.52 [0.25, 1.08] |
| Cleft palate | 5 (0.1%) | 291 (0.1%) | 1.03 [0.43, 2.5] | 0.76 [0.31, 1.84] | 0.21 [0.07, 0.6] |
| Gastrointestinal | 14 (0.3%) | 754 (0.3%) | 1.12 [0.66, 1.89] | 1.15 [0.67, 1.96] | 1.16 [0.46, 2.95] |
| Genitourinary | 60 (1.4%) | 3,340 (1.3%) | 1.08 [0.84, 1.39] | 1.07 [0.82, 1.4] | 1.16 [0.69, 1.94] |

Models were matched and adjusted for maternal age, ethnicity, lack of perinatal care, diabetes, obesity, folic acid supplementation, gravidity, calendar year, smoking, NSAID indication, and exposure to other analgesics or antipyretics.

### Table C6 – Adjusted associations between total Defined Daily Dose (DDD) of Diclofenac dispensed during the first trimester and overall risk of major malformations

| Total DDD for Diclofenac | Major Congenital Malformations | Adjusted RR (%95 CI) |
| --- | --- | --- |
| None | 18475/261231 (7.1%) | Reference |
| 1-7 | 184/2012 (9.1%) | 1.15 [0.99, 1.33] |
| 8-21 | 161/1867 (8.6%) | 1.09 [0.93, 1.27] |
| >21 | 2/33 (6.1%) | 0.81 [0.2, 3.23] |

Models were adjusted for maternal age, ethnicity, lack of perinatal care, diabetes, obesity, folic acid supplementation, gravidity, calendar year, smoking, NSAID indication, and exposure to other analgesics or antipyretics.

## Naproxen

### Table C7 – Comparison of maternal and pregnancy characteristics between pregnancies exposed and unexposed to Naproxen during the first trimester.

| **Characteristic** | **Naproxen** N = 3,102*^1^* | **Unexposed** N = 261,756*^1^* | **p-value***^2^* | **aSMD before** | **aSMD after** |
| --- | --- | --- | --- | --- | --- |
| **Calendar year of birth*^*^*** |  |  | <0.001 | 0.16 | 0.07 |
| 1998–2002 | 695 (22%) | 58,627 (22%) |  |  |  |
| 2003–2007 | 949 (31%) | 61,043 (23%) |  |  |  |
| 2008–2012 | 836 (27%) | 66,356 (25%) |  |  |  |
| 2013–2017 | 622 (20%) | 75,730 (29%) |  |  |  |
| **Maternal delivery age, years*^*^*** |  |  | 0.4 | 0.01 | 0.01 |
| <20 | 85 (2.7%) | 5,663 (2.2%) |  |  |  |
| 20–24 | 642 (21%) | 53,692 (21%) |  |  |  |
| 25–29 | 941 (30%) | 81,085 (31%) |  |  |  |
| 30–34 | 800 (26%) | 68,834 (26%) |  |  |  |
| 35–39 | 488 (16%) | 39,657 (15%) |  |  |  |
| 40–44 | 141 (4.5%) | 12,310 (4.7%) |  |  |  |
| ≥45 | 5 (0.2%) | 515 (0.2%) |  |  |  |
| **Maternal ethnic group (Bedouin)*^*^*** | 2,709 (87%) | 143,505 (55%) | <0.001 | 0.77 | 0.09 |
| **Maternal obesity*^*^*** | 39 (1.3%) | 1,318 (0.5%) | <0.001 | 0.08 | 0.01 |
| **Maternal smoking during pregnancy*^*^*** | 40 (1.3%) | 991 (0.4%) | <0.001 | 0.1 | 0.01 |
| **Maternal diabetes*^*^*** | 39 (1.3%) | 954 (0.4%) | <0.001 | 0.1 | 0.02 |
| **Maternal comorbidity indicating NSAIDS Tx*^*^*** | 450 (15%) | 25,130 (9.6%) | <0.001 | 0.15 | 0.1 |
| **Maternal exposure to other antipyretics** | 1,917 (62%) | 42,901 (16%) | <0.001 | 1.05 | 0.03 |
| **Gravidity*^*^*** | 4 (2, 7) | 3 (2, 5) | <0.001 | 0.41 | 0 |
| **Pregnancy age, days** | 278 (266, 283) | 275 (266, 281) | <0.001 | 0.08 | 0.07 |
| Missing | 12 | 699 |  |  |  |
| **Conception by assisted reproductive technology** | 5 (0.2%) | 914 (0.3%) | 0.077 | 0.04 | 0.06 |
| **Conception by insemination** | 3 (<0.1%) | 182 (<0.1%) | 0.5 | 0.01 | 0.01 |
| **Conception by IVF** | 2 (<0.1%) | 791 (0.3%) | 0.016 | 0.06 | 0.07 |
| **Lack of prenatal care*^*^*** | 37 (1.2%) | 1,784 (0.7%) | <0.001 | 0.05 | 0.04 |
| **Folic acid*^*^*** | 1,382 (45%) | 41,910 (16%) | <0.001 | 0.65 | 0.11 |
| **Sex of newborn (males)** | 1,602 (52%) | 133,321 (51%) | 0.6 | 0.01 | 0.03 |
| Missing | 6 | 1,622 |  |  |  |
| **Pregnancy termination** | 6 (0.2%) | 1,622 (0.6%) | 0.003 | 0.07 | 0.08 |
| *^1^*n (%); Median (Q1, Q3) | | | | | |
| *^2^*Differences between groups were assessed using Wilcoxon rank sum test for continuous variables and Pearson's Chi-squared test for categorical variables. | | | | | |
| *^*^*Covariates included in adjusted models | | | | | |
| aSMD, absolute Standardized Mean Difference; ATE, Average Treatment Effect; ESS, Effective Sample Size; NSAIDS, Nonsteroidal anti-inflammatory drugs; Tx, Treatment. | | | | | |
| ESS for matching-adjusted model targeting the ATE were 545 exposed and 261,573 controls. No actual units were discarded during the proccess. | | | | | |
| Maternal obesity was defined using ICD-9 codes 278.0–278.4, diabetes using ICD-9 250.* and gestational diabetes (GDM) 648.00–648.04, and folic acid use as dispensation during the first trimester. Maternal smoking was based on self-report or ICD-9 code 305.1. Exposure to other analgesics or antipyretics was defined as first-trimester exposure to either acetaminophen or dipyrone. NSAID indications were defined as first-trimester diagnoses for conditions commonly treated with NSAIDs, including musculoskeletal/joint disorders, pain and inflammatory conditions, injuries/fractures, and pregnancy-related diagnoses such as threatened abortion or fever/infection. Gravidity refers to total prior pregnancies. | | | | | |
| Missing values for fetal sex reflect pregnancy terminations due to suspected fetal malformations, for which fetal sex could not be determined. | | | | | |
| SMDs for *Calendar year of birth* were calculated using the ordinal calendar year variable; grouped categories are shown for presentation purposes only. | | | | | |

### Table C8 – Crude, adjusted, and matched relative risks for Naproxen exposure in relation to major congenital malformations overall and by specific organ systems

| Malformation | Naproxen N = 3,102 | Unexposed N = 261,756 | Unadjusted | Adjusted | Matched Adjusted |
| --- | --- | --- | --- | --- | --- |
| Major congenital malformations | 268 (8.6%) | 18,450 (7.0%) | 1.23 [1.09, 1.38] | 1.08 [0.95, 1.24] | 0.97 [0.73, 1.3] |
| Cardiovascular | 140 (4.5%) | 8,602 (3.3%) | 1.37 [1.16, 1.62] | 1.11 [0.92, 1.33] | 1.33 [0.88, 2] |
| Musculoskeletal | 33 (1.1%) | 2,839 (1.1%) | 0.98 [0.7, 1.38] | 0.88 [0.61, 1.29] | 0.59 [0.35, 1.02] |
| Central nervous system | 23 (0.7%) | 1,483 (0.6%) | 1.31 [0.87, 1.98] | 1.05 [0.69, 1.61] | 0.61 [0.33, 1.15] |
| Cleft palate | 5 (0.2%) | 291 (0.1%) | 1.45 [0.6, 3.51] | 1.07 [0.44, 2.59] | 0.3 [0.11, 0.79] |
| Gastrointestinal | 7 (0.2%) | 761 (0.3%) | 0.78 [0.37, 1.63] | 0.8 [0.38, 1.68] | 0.9 [0.24, 3.3] |
| Genitourinary | 40 (1.3%) | 3,360 (1.3%) | 1 [0.74, 1.37] | 1 [0.7, 1.41] | 0.61 [0.34, 1.09] |

Models were matched and adjusted for maternal age, ethnicity, lack of perinatal care, diabetes, obesity, folic acid supplementation, gravidity, calendar year, smoking, NSAID indication, and exposure to other analgesics or antipyretics.

### Table C9 – Adjusted associations between total Defined Daily Dose (DDD) of Naproxen dispensed during the first trimester and overall risk of major malformations

| Total DDD for Naproxen | Major Congenital Malformations | Adjusted RR (%95 CI) |
| --- | --- | --- |
| None | 18,626/262,864 (7.1%) | Reference |
| 1-7 | 36/462 (7.8%) | 0.97 [0.7, 1.34] |
| 8-21 | 149/1,668 (8.9%) | 1.11 [0.94, 1.31] |
| >21 | 11/149 (7.4%) | 0.93 [0.52, 1.69] |

Models were adjusted for maternal age, ethnicity, lack of perinatal care, diabetes, obesity, folic acid supplementation, gravidity, calendar year, smoking, NSAID indication, and exposure to other analgesics or antipyretics.

## Indomethacin

### Table C10 – Comparison of maternal and pregnancy characteristics between pregnancies exposed and unexposed to Indomethacin during the first trimester.

| **Characteristic** | **Indometacin** N = 287*^1^* | **Unexposed** N = 264,571*^1^* | **p-value***^2^* | **aSMD before** | **aSMD after** |
| --- | --- | --- | --- | --- | --- |
| **Calendar year of birth*^*^*** |  |  | <0.001 | 0.33 | 0.19 |
| 1998–2002 | 78 (27%) | 59,244 (22%) |  |  |  |
| 2003–2007 | 95 (33%) | 61,897 (23%) |  |  |  |
| 2008–2012 | 74 (26%) | 67,118 (25%) |  |  |  |
| 2013–2017 | 40 (14%) | 76,312 (29%) |  |  |  |
| **Maternal delivery age, years*^*^*** |  |  |  | 0.23 | 0.12 |
| <20 | 0 (0%) | 5,748 (2.2%) |  |  |  |
| 20–24 | 42 (15%) | 54,292 (21%) |  |  |  |
| 25–29 | 84 (29%) | 81,942 (31%) |  |  |  |
| 30–34 | 85 (30%) | 69,549 (26%) |  |  |  |
| 35–39 | 59 (21%) | 40,086 (15%) |  |  |  |
| 40–44 | 15 (5.2%) | 12,436 (4.7%) |  |  |  |
| ≥45 | 2 (0.7%) | 518 (0.2%) |  |  |  |
| **Maternal ethnic group (Bedouin)*^*^*** | 227 (79%) | 145,987 (55%) | <0.001 | 0.53 | 0.09 |
| **Maternal obesity*^*^*** | 20 (7.0%) | 1,337 (0.5%) | <0.001 | 0.35 | 0 |
| **Maternal smoking during pregnancy*^*^*** | 1 (0.3%) | 1,030 (0.4%) | >0.9 | 0.01 | 0.01 |
| **Maternal diabetes*^*^*** | 8 (2.8%) | 985 (0.4%) | <0.001 | 0.19 | 0.02 |
| **Maternal comorbidity indicating NSAIDS Tx*^*^*** | 72 (25%) | 25,508 (9.6%) | <0.001 | 0.42 | 0.07 |
| **Maternal exposure to other antipyretics** | 192 (67%) | 44,626 (17%) | <0.001 | 1.18 | 0.02 |
| **Gravidity*^*^*** | 5 (3, 8) | 3 (2, 5) | <0.001 | 0.55 | 0.14 |
| **Pregnancy age, days** | 274 (266, 280) | 275 (266, 281) | 0.2 | 0.07 | 0.1 |
| Missing | 1 | 710 |  |  |  |
| **Conception by assisted reproductive technology** | 4 (1.4%) | 915 (0.3%) | 0.018 | 0.11 | 0.09 |
| **Conception by insemination** | 0 (0%) | 185 (<0.1%) | >0.9 | 0.04 | 0.04 |
| **Conception by IVF** | 4 (1.4%) | 789 (0.3%) | 0.011 | 0.12 | 0.1 |
| **Lack of prenatal care*^*^*** | 1 (0.3%) | 1,820 (0.7%) | >0.9 | 0.05 | 0.09 |
| **Folic acid*^*^*** | 120 (42%) | 43,172 (16%) | <0.001 | 0.58 | 0.16 |
| **Sex of newborn (males)** | 155 (54%) | 134,768 (51%) | 0.4 | 0.06 | 0.04 |
| Missing | 0 | 1,628 |  |  |  |
| **Pregnancy termination** | 0 (0%) | 1,628 (0.6%) | 0.4 | 0.11 | 0.11 |
| *^1^*n (%); Median (Q1, Q3) | | | | | |
| *^2^*Differences between groups were assessed using Wilcoxon rank sum test for continuous variables and Pearson's Chi-squared test for categorical variables. | | | | | |
| *^*^*Covariates included in adjusted models | | | | | |
| aSMD, absolute Standardized Mean Difference; ATE, Average Treatment Effect; ESS, Effective Sample Size; NSAIDS, Nonsteroidal anti-inflammatory drugs; Tx, Treatment. | | | | | |
| ESS for matching-adjusted model targeting the ATE were 50 exposed and 264,567 controls. No actual units were discarded during the proccess. | | | | | |
| Maternal obesity was defined using ICD-9 codes 278.0–278.4, diabetes using ICD-9 250.* and gestational diabetes (GDM) 648.00–648.04, and folic acid use as dispensation during the first trimester. Maternal smoking was based on self-report or ICD-9 code 305.1. Exposure to other analgesics or antipyretics was defined as first-trimester exposure to either acetaminophen or dipyrone. NSAID indications were defined as first-trimester diagnoses for conditions commonly treated with NSAIDs, including musculoskeletal/joint disorders, pain and inflammatory conditions, injuries/fractures, and pregnancy-related diagnoses such as threatened abortion or fever/infection. Gravidity refers to total prior pregnancies. | | | | | |
| Missing values for fetal sex reflect pregnancy terminations due to suspected fetal malformations, for which fetal sex could not be determined. | | | | | |
| SMDs for *Calendar year of birth* were calculated using the ordinal calendar year variable; grouped categories are shown for presentation purposes only. | | | | | |

### Table C11 – Crude, adjusted, and matched relative risks for Indomethacin exposure in relation to major congenital malformations overall and by specific organ systems

| Malformation | Indomethacin N = 287 | Unexposed N = 264,571 | Unadjusted | Adjusted | Matched Adjusted |
| --- | --- | --- | --- | --- | --- |
| Major congenital malformations | 26 (9.1%) | 18,692 (7.1%) | 1.28 [0.87, 1.88] | 1.13 [0.79, 1.61] | 1.76 [0.59, 5.21] |
| Cardiovascular | 12 (4.2%) | 8,730 (3.3%) | 1.27 [0.72, 2.23] | 0.99 [0.59, 1.68] | 0.74 [0.27, 1.99] |
| Musculoskeletal | 2 (0.7%) | 2,870 (1.1%) | 0.64 [0.16, 2.57] | 0.61 [0.15, 2.4] | 1.02 [0.25, 4.2] |
| Central nervous system | 2 (0.7%) | 1,504 (0.6%) | 1.23 [0.31, 4.91] | 1.06 [0.27, 4.16] | 0.14 [0.02, 0.82] |
| Cleft palate | 0 (0%) | 296 (0.1%) | 0 [0, 4.77e+169] | 0 [0, 0] | 0 [0, 0] |
| Gastrointestinal | 3 (1.0%) | 765 (0.3%) | 3.62 [1.16, 11.2] | 3.77 [0.91, 15.7] | 0.52 [0.12, 2.29] |
| Genitourinary | 5 (1.7%) | 3,395 (1.3%) | 1.36 [0.56, 3.26] | 1.38 [0.5, 3.84] | 6.27 [1.17, 33.6] |

Models were matched and adjusted for maternal age, ethnicity, lack of perinatal care, diabetes, obesity, folic acid supplementation, gravidity, calendar year, smoking, NSAID indication, and exposure to other analgesics or antipyretics.

### Table C12 – Adjusted associations between total Defined Daily Dose (DDD) of Indomethacin dispensed during the first trimester and overall risk of major malformations

| Total DDD for Indomethacin | Major Congenital Malformations | Adjusted RR (%95 CI) |
| --- | --- | --- |
| None | 18,797/264,862 (7.1%) | Reference |
| 1-7 | 12/141 (8.5%) | 1.05 [0.6, 1.86] |
| 8-21 | 13/140 (9.3%) | 1.17 [0.68, 2.02] |

Models were adjusted for maternal age, ethnicity, lack of perinatal care, diabetes, obesity, folic acid supplementation, gravidity, calendar year, smoking, NSAID indication, and exposure to other analgesics or antipyretics.

## Etodolac

### Table C13 – Comparison of maternal and pregnancy characteristics between pregnancies exposed and unexposed to Etodolac during the first trimester.

| **Characteristic** | **Etodolac** N = 1,440*^1^* | **Unexposed** N = 263,418*^1^* | **p-value***^2^* | **aSMD before** | **aSMD after** |
| --- | --- | --- | --- | --- | --- |
| **Calendar year of birth*^*^*** |  |  | <0.001 | 0.07 | 0.01 |
| 1998–2002 | 245 (17%) | 59,077 (22%) |  |  |  |
| 2003–2007 | 362 (25%) | 61,630 (23%) |  |  |  |
| 2008–2012 | 455 (32%) | 66,737 (25%) |  |  |  |
| 2013–2017 | 378 (26%) | 75,974 (29%) |  |  |  |
| **Maternal delivery age, years*^*^*** |  |  |  | 0.07 | 0 |
| <20 | 33 (2.3%) | 5,715 (2.2%) |  |  |  |
| 20–24 | 322 (22%) | 54,012 (21%) |  |  |  |
| 25–29 | 476 (33%) | 81,550 (31%) |  |  |  |
| 30–34 | 352 (24%) | 69,282 (26%) |  |  |  |
| 35–39 | 195 (14%) | 39,950 (15%) |  |  |  |
| 40–44 | 58 (4.0%) | 12,393 (4.7%) |  |  |  |
| ≥45 | 4 (0.3%) | 516 (0.2%) |  |  |  |
| **Maternal ethnic group (Bedouin)*^*^*** | 1,159 (80%) | 145,055 (55%) | <0.001 | 0.57 | 0.06 |
| **Maternal obesity*^*^*** | 25 (1.7%) | 1,332 (0.5%) | <0.001 | 0.12 | 0 |
| **Maternal smoking during pregnancy*^*^*** | 9 (0.6%) | 1,022 (0.4%) | 0.15 | 0.03 | 0.02 |
| **Maternal diabetes*^*^*** | 32 (2.2%) | 961 (0.4%) | <0.001 | 0.16 | 0.01 |
| **Maternal comorbidity indicating NSAIDS Tx*^*^*** | 232 (16%) | 25,348 (9.6%) | <0.001 | 0.19 | 0.02 |
| **Maternal exposure to other antipyretics** | 867 (60%) | 43,951 (17%) | <0.001 | 1 | 0.01 |
| **Gravidity*^*^*** | 4 (2, 6) | 3 (2, 5) | <0.001 | 0.22 | 0.08 |
| **Pregnancy age, days** | 275 (266, 280) | 275 (266, 281) | >0.9 | 0.02 | 0.08 |
| Missing | 3 | 708 |  |  |  |
| **Conception by assisted reproductive technology** | 24 (1.7%) | 895 (0.3%) | <0.001 | 0.13 | 0.1 |
| **Conception by insemination** | 0 (0%) | 185 (<0.1%) | 0.6 | 0.04 | 0.04 |
| **Conception by IVF** | 24 (1.7%) | 769 (0.3%) | <0.001 | 0.14 | 0.1 |
| **Lack of prenatal care*^*^*** | 11 (0.8%) | 1,810 (0.7%) | 0.7 | 0.01 | 0.01 |
| **Folic acid*^*^*** | 645 (45%) | 42,647 (16%) | <0.001 | 0.65 | 0.09 |
| **Sex of newborn (males)** | 732 (51%) | 134,191 (51%) | 0.8 | 0.01 | 0.05 |
| Missing | 4 | 1,624 |  |  |  |
| **Pregnancy termination** | 4 (0.3%) | 1,624 (0.6%) | 0.10 | 0.05 | 0.07 |
| *^1^*n (%); Median (Q1, Q3) | | | | | |
| *^2^*Differences between groups were assessed using Wilcoxon rank sum test for continuous variables and Pearson's Chi-squared test for categorical variables. | | | | | |
| *^*^*Covariates included in adjusted models | | | | | |
| aSMD, absolute Standardized Mean Difference; ATE, Average Treatment Effect; ESS, Effective Sample Size; NSAIDS, Nonsteroidal anti-inflammatory drugs; Tx, Treatment. | | | | | |
| ESS for matching-adjusted model targeting the ATE were 366 exposed and 263,388 controls. No actual units were discarded during the proccess. | | | | | |
| Maternal obesity was defined using ICD-9 codes 278.0–278.4, diabetes using ICD-9 250.* and gestational diabetes (GDM) 648.00–648.04, and folic acid use as dispensation during the first trimester. Maternal smoking was based on self-report or ICD-9 code 305.1. Exposure to other analgesics or antipyretics was defined as first-trimester exposure to either acetaminophen or dipyrone. NSAID indications were defined as first-trimester diagnoses for conditions commonly treated with NSAIDs, including musculoskeletal/joint disorders, pain and inflammatory conditions, injuries/fractures, and pregnancy-related diagnoses such as threatened abortion or fever/infection. Gravidity refers to total prior pregnancies. | | | | | |
| Missing values for fetal sex reflect pregnancy terminations due to suspected fetal malformations, for which fetal sex could not be determined. | | | | | |
| SMDs for *Calendar year of birth* were calculated using the ordinal calendar year variable; grouped categories are shown for presentation purposes only. | | | | | |

### Table C14 – Crude, adjusted, and matched relative risks for Etodolac exposure in relation to major congenital malformations overall and by specific organ systems

| Malformation | Etodolac N = 1,440 | Unexposed N = 263,418 | Unadjusted | Adjusted | Matched Adjusted |
| --- | --- | --- | --- | --- | --- |
| Major congenital malformations | 123 (8.5%) | 18,595 (7.1%) | 1.21 [1.01, 1.44] | 1.08 [0.89, 1.3] | 0.88 [0.64, 1.21] |
| Cardiovascular | 74 (5.1%) | 8,668 (3.3%) | 1.56 [1.24, 1.96] | 1.27 [0.99, 1.63] | 0.86 [0.59, 1.25] |
| Musculoskeletal | 17 (1.2%) | 2,855 (1.1%) | 1.09 [0.68, 1.75] | 0.97 [0.59, 1.58] | 0.82 [0.39, 1.71] |
| Central nervous system | 6 (0.4%) | 1,500 (0.6%) | 0.73 [0.33, 1.63] | 0.6 [0.27, 1.33] | 0.71 [0.25, 1.99] |
| Cleft palate | 2 (0.1%) | 294 (0.1%) | 1.24 [0.31, 5] | 0.96 [0.24, 3.85] | 1.25 [0.21, 7.44] |
| Gastrointestinal | 2 (0.1%) | 766 (0.3%) | 0.48 [0.12, 1.91] | 0.47 [0.12, 1.84] | 0.17 [0.04, 0.71] |
| Genitourinary | 19 (1.3%) | 3,381 (1.3%) | 1.03 [0.65, 1.61] | 0.98 [0.59, 1.63] | 0.68 [0.26, 1.74] |

Models were matched and adjusted for maternal age, ethnicity, lack of perinatal care, diabetes, obesity, folic acid supplementation, gravidity, calendar year, smoking, NSAID indication, and exposure to other analgesics or antipyretics.

### Table C15 – Adjusted associations between total Defined Daily Dose (DDD) of Etodolac dispensed during the first trimester and overall risk of major malformations

| Total DDD for Etodolac | Major Congenital Malformations | Adjusted RR (%95 CI) |
| --- | --- | --- |
| None | 18,700/263,709 (7.1%) | Reference |
| 1-7 | 2/20 (10.0%) | 1.21 [0.3, 4.85] |
| 8-21 | 80/1,070 (7.5%) | 0.94 [0.75, 1.17] |
| >21 | 40/344 (11.6%) | 1.47 [1.08, 2] |

Models were adjusted for maternal age, ethnicity, lack of perinatal care, diabetes, obesity, folic acid supplementation, gravidity, calendar year, smoking, NSAID indication, and exposure to other analgesics or antipyretics.

## Piroxicam

### Table C16 – Comparison of maternal and pregnancy characteristics between pregnancies exposed and unexposed to Piroxicam during the first trimester.

| **Characteristic** | **Piroxicam** N = 91*^1^* | **Unexposed** N = 264,767*^1^* | **p-value***^2^* | **aSMD before** | **aSMD after** |
| --- | --- | --- | --- | --- | --- |
| **Calendar year of birth*^*^*** |  |  | 0.027 | 0.35 | 0.3 |
| 1998–2002 | 11 (12%) | 59,311 (22%) |  |  |  |
| 2003–2007 | 17 (19%) | 61,975 (23%) |  |  |  |
| 2008–2012 | 28 (31%) | 67,164 (25%) |  |  |  |
| 2013–2017 | 35 (38%) | 76,317 (29%) |  |  |  |
| **Maternal delivery age, years*^*^*** |  |  |  | 0.02 | 0.25 |
| <20 | 4 (4.4%) | 5,744 (2.2%) |  |  |  |
| 20–24 | 16 (18%) | 54,318 (21%) |  |  |  |
| 25–29 | 31 (34%) | 81,995 (31%) |  |  |  |
| 30–34 | 25 (27%) | 69,609 (26%) |  |  |  |
| 35–39 | 10 (11%) | 40,135 (15%) |  |  |  |
| 40–44 | 5 (5.5%) | 12,446 (4.7%) |  |  |  |
| ≥45 | 0 (0%) | 520 (0.2%) |  |  |  |
| **Maternal ethnic group (Bedouin)*^*^*** | 75 (82%) | 146,139 (55%) | <0.001 | 0.61 | 0.15 |
| **Maternal obesity*^*^*** | 8 (8.8%) | 1,349 (0.5%) | <0.001 | 0.4 | 0.02 |
| **Maternal smoking during pregnancy*^*^*** | 0 (0%) | 1,031 (0.4%) | >0.9 | 0.09 | 0.09 |
| **Maternal diabetes*^*^*** | 11 (12%) | 982 (0.4%) | <0.001 | 0.5 | 0.01 |
| **Maternal comorbidity indicating NSAIDS Tx*^*^*** | 13 (14%) | 25,567 (9.7%) | 0.13 | 0.14 | 0.35 |
| **Maternal exposure to other antipyretics** | 51 (56%) | 44,767 (17%) | <0.001 | 0.89 | 0.04 |
| **Gravidity*^*^*** | 4 (2, 6) | 3 (2, 5) | 0.044 | 0.21 | 0.15 |
| **Pregnancy age, days** | 274 (266, 280) | 275 (266, 281) | 0.6 | 0.09 | 0.01 |
| Missing | 0 | 711 |  |  |  |
| **Conception by assisted reproductive technology** | 0 (0%) | 919 (0.3%) | >0.9 | 0.08 | 0.08 |
| **Conception by insemination** | 0 (0%) | 185 (<0.1%) | >0.9 | 0.04 | 0.04 |
| **Conception by IVF** | 0 (0%) | 793 (0.3%) | >0.9 | 0.08 | 0.08 |
| **Lack of prenatal care*^*^*** | 0 (0%) | 1,821 (0.7%) | >0.9 | 0.12 | 0.12 |
| **Folic acid*^*^*** | 43 (47%) | 43,249 (16%) | <0.001 | 0.7 | 0.34 |
| **Sex of newborn (males)** | 37 (41%) | 134,886 (51%) | 0.043 | 0.21 | 0 |
| Missing | 0 | 1,628 |  |  |  |
| **Pregnancy termination** | 0 (0%) | 1,628 (0.6%) | >0.9 | 0.11 | 0.11 |
| *^1^*n (%); Median (Q1, Q3) | | | | | |
| *^2^*Differences between groups were assessed using Wilcoxon rank sum test for continuous variables and Pearson's Chi-squared test for categorical variables. | | | | | |
| *^*^*Covariates included in adjusted models | | | | | |
| aSMD, absolute Standardized Mean Difference; ATE, Average Treatment Effect; ESS, Effective Sample Size; NSAIDS, Nonsteroidal anti-inflammatory drugs; Tx, Treatment. | | | | | |
| ESS for matching-adjusted model targeting the ATE were 24 exposed and 264,765 controls. No actual units were discarded during the proccess. | | | | | |
| Maternal obesity was defined using ICD-9 codes 278.0–278.4, diabetes using ICD-9 250.* and gestational diabetes (GDM) 648.00–648.04, and folic acid use as dispensation during the first trimester. Maternal smoking was based on self-report or ICD-9 code 305.1. Exposure to other analgesics or antipyretics was defined as first-trimester exposure to either acetaminophen or dipyrone. NSAID indications were defined as first-trimester diagnoses for conditions commonly treated with NSAIDs, including musculoskeletal/joint disorders, pain and inflammatory conditions, injuries/fractures, and pregnancy-related diagnoses such as threatened abortion or fever/infection. Gravidity refers to total prior pregnancies. | | | | | |
| Missing values for fetal sex reflect pregnancy terminations due to suspected fetal malformations, for which fetal sex could not be determined. | | | | | |
| SMDs for *Calendar year of birth* were calculated using the ordinal calendar year variable; grouped categories are shown for presentation purposes only. | | | | | |

### Table C17 – Crude, adjusted, and matched relative risks for Piroxicam exposure in relation to major congenital malformations overall and by specific organ systems

| Malformation | Piroxicam N = 91 | Unexposed N = 264,767 | Unadjusted | Adjusted | Matched Adjusted |
| --- | --- | --- | --- | --- | --- |
| Major congenital malformations | 10 (11%) | 18,708 (7.1%) | 1.56 [0.84, 2.89] | 1.32 [0.82, 2.12] | 1.47 [1.07, 2.03] |
| Cardiovascular | 3 (3.3%) | 8,739 (3.3%) | 1 [0.32, 3.1] | 0.78 [0.3, 2] | 0.51 [0.36, 0.74] |
| Musculoskeletal | 3 (3.3%) | 2,869 (1.1%) | 3.04 [0.98, 9.44] | 2.5 [0.88, 7.12] | 1.64 [1, 2.69] |
| Central nervous system | 1 (1.1%) | 1,505 (0.6%) | 1.93 [0.27, 13.7] | 1.43 [0.19, 10.6] | 0.49 [0.19, 1.3] |
| Cleft palate | 0 (0%) | 296 (0.1%) | 0 [0, 3.58e+183] | 0 [0, 0] | 0 [0, 0] |
| Gastrointestinal | 0 (0%) | 768 (0.3%) | 0 [0, 5.33e+109] | 0 [0, 0] | 0 [0, 0] |
| Genitourinary | 2 (2.2%) | 3,398 (1.3%) | 1.71 [0.43, 6.85] | 1.5 [0.47, 4.84] | 0.03 [0.01, 0.05] |

Models were matched and adjusted for maternal age, ethnicity, lack of perinatal care, diabetes, obesity, folic acid supplementation, gravidity, calendar year, smoking, NSAID indication, and exposure to other analgesics or antipyretics.

### Table C18 – Adjusted associations between total Defined Daily Dose (DDD) of Piroxicam dispensed during the first trimester and overall risk of major malformations

| Total DDD for Piroxicam | Major Congenital Malformations | Adjusted RR (%95 CI) |
| --- | --- | --- |
| None | 18,812/265052 (7.1%) | Reference |
| 8-21 | 10/89 (11.2%) | 1.35 [0.72, 2.51] |
| >21 | 0/2 (0.0%) | 0 [0, 1.96587176882029e+35] |

Models were adjusted for maternal age, ethnicity, lack of perinatal care, diabetes, obesity, folic acid supplementation, gravidity, calendar year, smoking, NSAID indication, and exposure to other analgesics or antipyretics.

## Lornoxicam

### Table C19 – Comparison of maternal and pregnancy characteristics between pregnancies exposed and unexposed to Lornoxicam during the first trimester.

| **Characteristic** | **Lornoxicam** N = 62*^1^* | **Unexposed** N = 264,796*^1^* | **p-value***^2^* | **aSMD before** | **aSMD after** |
| --- | --- | --- | --- | --- | --- |
| **Calendar year of birth*^*^*** |  |  | 0.002 | 0.46 | 0.4 |
| 1998–2002 | 4 (6.5%) | 59,318 (22%) |  |  |  |
| 2003–2007 | 10 (16%) | 61,982 (23%) |  |  |  |
| 2008–2012 | 25 (40%) | 67,167 (25%) |  |  |  |
| 2013–2017 | 23 (37%) | 76,329 (29%) |  |  |  |
| **Maternal delivery age, years*^*^*** |  |  |  | 0.33 | 0.44 |
| <20 | 1 (1.6%) | 5,747 (2.2%) |  |  |  |
| 20–24 | 18 (29%) | 54,316 (21%) |  |  |  |
| 25–29 | 23 (37%) | 82,003 (31%) |  |  |  |
| 30–34 | 15 (24%) | 69,619 (26%) |  |  |  |
| 35–39 | 3 (4.8%) | 40,142 (15%) |  |  |  |
| 40–44 | 2 (3.2%) | 12,449 (4.7%) |  |  |  |
| ≥45 | 0 (0%) | 520 (0.2%) |  |  |  |
| **Maternal ethnic group (Bedouin)*^*^*** | 52 (84%) | 146,162 (55%) | <0.001 | 0.66 | 0.05 |
| **Maternal obesity*^*^*** | 0 (0%) | 1,357 (0.5%) | >0.9 | 0.1 | 0.1 |
| **Maternal smoking during pregnancy*^*^*** | 0 (0%) | 1,031 (0.4%) | >0.9 | 0.09 | 0.09 |
| **Maternal diabetes*^*^*** | 0 (0%) | 993 (0.4%) | >0.9 | 0.09 | 0.09 |
| **Maternal comorbidity indicating NSAIDS Tx*^*^*** | 15 (24%) | 25,565 (9.7%) | <0.001 | 0.4 | 0.1 |
| **Maternal exposure to other antipyretics** | 30 (48%) | 44,788 (17%) | <0.001 | 0.71 | 0.14 |
| **Gravidity*^*^*** | 4 (2, 6) | 3 (2, 5) | 0.2 | 0.11 | 0.43 |
| **Pregnancy age, days** | 274 (266, 280) | 275 (266, 281) | >0.9 | 0.12 | 0.18 |
| Missing | 0 | 711 |  |  |  |
| **Conception by assisted reproductive technology** | 0 (0%) | 919 (0.3%) | >0.9 | 0.08 | 0.08 |
| **Conception by insemination** | 0 (0%) | 185 (<0.1%) | >0.9 | 0.04 | 0.04 |
| **Conception by IVF** | 0 (0%) | 793 (0.3%) | >0.9 | 0.08 | 0.08 |
| **Lack of prenatal care*^*^*** | 0 (0%) | 1,821 (0.7%) | >0.9 | 0.12 | 0.12 |
| **Folic acid*^*^*** | 26 (42%) | 43,266 (16%) | <0.001 | 0.59 | 0.1 |
| **Sex of newborn (males)** | 31 (50%) | 134,892 (51%) | 0.8 | 0.03 | 0.1 |
| Missing | 0 | 1,628 |  |  |  |
| **Pregnancy termination** | 0 (0%) | 1,628 (0.6%) | >0.9 | 0.11 | 0.11 |
| *^1^*n (%); Median (Q1, Q3) | | | | | |
| *^2^*Differences between groups were assessed using Wilcoxon rank sum test for continuous variables and Pearson's Chi-squared test for categorical variables. | | | | | |
| *^*^*Covariates included in adjusted models | | | | | |
| aSMD, absolute Standardized Mean Difference; ATE, Average Treatment Effect; ESS, Effective Sample Size; NSAIDS, Nonsteroidal anti-inflammatory drugs; Tx, Treatment. | | | | | |
| ESS for matching-adjusted model targeting the ATE were 14 exposed and 264,796 controls. No actual units were discarded during the proccess. | | | | | |
| Maternal obesity was defined using ICD-9 codes 278.0–278.4, diabetes using ICD-9 250.* and gestational diabetes (GDM) 648.00–648.04, and folic acid use as dispensation during the first trimester. Maternal smoking was based on self-report or ICD-9 code 305.1. Exposure to other analgesics or antipyretics was defined as first-trimester exposure to either acetaminophen or dipyrone. NSAID indications were defined as first-trimester diagnoses for conditions commonly treated with NSAIDs, including musculoskeletal/joint disorders, pain and inflammatory conditions, injuries/fractures, and pregnancy-related diagnoses such as threatened abortion or fever/infection. Gravidity refers to total prior pregnancies. | | | | | |
| Missing values for fetal sex reflect pregnancy terminations due to suspected fetal malformations, for which fetal sex could not be determined. | | | | | |
| SMDs for *Calendar year of birth* were calculated using the ordinal calendar year variable; grouped categories are shown for presentation purposes only. | | | | | |

### Table C20 – Crude, adjusted, and matched relative risks for Lornoxicam exposure in relation to major congenital malformations overall and by specific organ systems

Models were matched and adjusted for maternal age, ethnicity, lack of perinatal care, diabetes, obesity, folic acid supplementation, gravidity, calendar year, smoking, NSAID indication, and exposure to other analgesics or antipyretics.

| Malformation | Lornoxicam N = 62 | Unexposed N = 264,796 | Unadjusted | Adjusted | Matched Adjusted |
| --- | --- | --- | --- | --- | --- |
| Major congenital malformations | 8 (13%) | 18,710 (7.1%) | 1.83 [0.91, 3.65] | 1.61 [0.89, 2.91] | 0.49 [0.32, 0.74] |
| Cardiovascular | 7 (11%) | 8,735 (3.3%) | 3.42 [1.63, 7.18] | 2.69 [1.54, 4.69] | 0.86 [0.63, 1.18] |
| Musculoskeletal | 1 (1.6%) | 2,871 (1.1%) | 1.49 [0.21, 10.6] | 1.3 [0.2, 8.6] | 0.45 [0.28, 0.73] |
| Central nervous system | 0 (0%) | 1,506 (0.6%) | 0 [0, 4.80e+79] | 0 [0, 0] | 0 [0, 0] |
| Cleft palate | 0 (0%) | 296 (0.1%) | 0 [0, 1.78e+223] | 0 [0, 0] | 0 [0, 0] |
| Gastrointestinal | 0 (0%) | 768 (0.3%) | 0 [0, 6.37e+133] | 0 [0, 0] | 0 [0, 0] |
| Genitourinary | 1 (1.6%) | 3,399 (1.3%) | 1.26 [0.18, 8.92] | 1.17 [0.18, 7.82] | 0.36 [0.18, 0.75] |

### Table C21 – Adjusted associations between total Defined Daily Dose (DDD) of Lornoxicam dispensed during the first trimester and overall risk of major malformations

| Total DDD for Lornoxicam | Major Congenital Malformations | Adjusted RR (%95 CI) |
| --- | --- | --- |
| None | 18,814/265086 (7.1%) | Reference |
| 1-7 | 1/22 (4.5%) | 0.58 [0.08, 4.14] |
| 8-21 | 7/35 (20.0%) | 2.43 [1.16, 5.1] |

Models were adjusted for maternal age, ethnicity, lack of perinatal care, diabetes, obesity, folic acid supplementation, gravidity, calendar year, smoking, NSAID indication, and exposure to other analgesics or antipyretics.

# Chapter 4 – STROBE checklist

## Appendix Checklist D1: STROBE Statement: checklist of items that should be included in reports of observational studies

First Trimester Nonsteroidal Anti-inflammatory Drugs Exposure and Risk of Major Congenital Malformations: a retrospective register-based cohort study

Ariel Avraham Hasidim^1,2,3^ MD, MPH, Itamar Ben Shitrit*^1,4,9^ MD, MPH, Daphna Idan^1,4^, MD, MPH, Tal Michael^1^ MD, MPH, PhD, Amalia Levy^1^ MPH, PhD , Gali Pariente^6^ MD, Eitan Lunenfeld MD, MHA^7^, Sharon Daniel MD, MPH, PhD^1,5,8^

1. Department of Epidemiology, Biostatistics, and Community Health Sciences, school of Public Health , Faculty of Health Sciences, Ben-Gurion University of the Negev ,Beer-Sheva, Israel.
2. Department of Pediatrics A, Schneider Children’s Medical Center of Israel, Petah Tikva 94903, Israel
3. Sackler Faculty of Medicine, Tel Aviv University, Tel Aviv 6997801, Israel
4. Joyce and Irving Goldman Medical School, Faculty of Health Sciences, Ben-Gurion University of the Negev, Beer-Sheva 8410501, Israel
5. Department of Pediatrics, Faculty of Health Sciences, Ben-Gurion University of the Negev ,Beer-Sheva, Israel.
6. Department of Obstetrics and Gynecology, Faculty of Health Sciences Ben-Gurion University of the Negev and Soroka University Medical Center ,Beer-Sheva, Isreal.
7. Adelson School of Medicine, Ariel University, Ariel, Israel
8. Clalit Health Services, Southern District, Beer-Sheva, Israel.
9. Clinical Research Center, Faculty of Health Sciences, Soroka University Medical Center, Ben-Gurion University of the Negev, Beer-Sheva, Israel

Corresponding Author:
Dr. Sharon Daniel
Ben Gurion University of the Negev
Beer-Sheva 84101, Israel
Tel: +972-509407722, E mail: daniels@post.bgu.ac.il
*Ariel Avraham Hasidim and Itamar Ben Shitrit should be accounted as “Equal Contribution Status”

|  | **Item No.** | **Recommendation** | **Location in manuscript where items are reported** | **Relevant text from manuscript** |
| --- | --- | --- | --- | --- |
| **Title and abstract** | 1 | (a) Indicate the study's design with a commonly used term in the title or the abstract | Title and abstract | Title: "First Trimester Nonsteroidal Anti-inflammatory Drugs Exposure and Risk of Major Congenital Malformations: a large population-based cohort of pregnancies" ...Abstract: "We conducted a population-based retrospective cohort study within the Southern Israeli Pregnancy Registry (siPREG) project" |
|  |  | (b) Provide in the abstract an informative and balanced summary of what was done and what was found | Abstract | "Background: Pain and fever are common in early pregnancy, yet their management poses a major clinical dilemma..." ... "Methods and Findings: We conducted a population-based retrospective cohort study..." ... "NSAIDs exposure, in total and as individual agents, was not associated with major congenital malformations overall (8.2% vs. 7.0%; matched aRR = 1.00 (95% CI [0.89,1.11]))" ... "The main limitation was the potential for minor exposure misclassification due to over-the-counter availability of ibuprofen" ... "Conclusion: In this large, population-based cohort, we found no evidence supporting an association between first-trimester exposure to NSAIDs and major congenital malformations" |
| ***Introduction*** | | | | |
| **Background/ rationale** | 2 | Explain the scientific background and rationale for the investigation being reported | Introduction, first, second and third paragraph | "During September 2025 regulatory advisories in the United States have raised concerns about potential adverse pregnancy outcomes associated with acetaminophen use during gestation" ... "Evidence suggest that a considerable number of pregnant women already avoid the use of analgesics due to concerns about fetal safety" ... "Inadequate analgetic management can cause significant maternal distress, increase the risk of perinatal depression, and potentially impair neonatal development" ... "The potential association between exposure to NSAIDs during the first trimester and the risk of major congenital malformations (MCMs) has been previously examined, however findings remain inconsistent" |
| **Objectives** | 3 | State specific objectives, including any prespecified hypotheses | Introduction, fourth paragraph | "This population-based cohort study was designed to assess the association between first trimester maternal exposure to NSAIDs, both overall and for specific drugs and an increased risk of MCMs. We hypothesized that any potential increase in risk would be modest." |
| ***Methods*** | | | | |
| **Study design** | 4 | Present key elements of study design early in the paper | Methods, Study Design. | "This study was conducted within the framework of the siPREG (Southern Israeli Pregnancy Registry), a population-based initiative investigating maternal and perinatal outcomes in southern Israel. The study complies with the Strengthening the Reporting of Observational Studies in Epidemiology (STROBE) reporting guidelines for observational studies and was approved by the Soroka University Medical Center (SUMC) ethics committee in accordance with the Declaration of Helsinki (approval number 0069–20-SOR; 7 March 2022)." |
| **Setting** | 5 | Describe the setting, locations, and relevant dates, including periods of recruitment, exposure, follow-up, and data collection | Methods, Setting and Participants. | "The cohort included all pregnancies of women aged 15–45 years insured by Clalit health services (CHS) maintenance organization in southern Israel that resulted in delivery or elective pregnancy termination for suspected fetal malformations at SUMC between 1998 and 2018. Clalit insures approximately 70% of women of reproductive age in the region, and SUMC accounts for nearly all deliveries (~98%) in the district" |
| **Participants** | 6 | (a) Cohort study—Give the eligibility criteria, and the sources and methods of selection of participants. Describe methods of follow-up | Methods, Setting and Participants and Outcomes sections. | "Pregnancies exposed to established teratogenic drugs (antimetabolites, isotretinoin, and anti-epileptic drugs), multiple gestations, or pregnancies with documented genetic or chromosomal diagnoses were excluded from the study. Analyses were restricted to pregnancies with complete data on model's covariates and outcomes." ... "MCMs were defined according to the Metropolitan Atlanta Congenital Defects Program (MACDP) of the Centers for Disease Control and Prevention and classified using the International Classification of Diseases, 9th Revision (ICD-9) by board certified neonatologists" |
|  |  | (b) Cohort study—For matched studies, give matching criteria and number of exposed and unexposed | Methods, Statistical Analysis, Results Table 1. | "generalized full matching was applied on the propensity score to further account for confounding, targeting the average treatment effect (ATE). The propensity score was estimated using a probit regression of the treatment on the covariates and achieved adequate balance. Matching weights were derived from this procedure and incorporated into the outcome model (no units were discarded by the matching)." ... "264,858 complete cases were included in the final cohort...20,202 pregnancies (7.6%) were exposed to NSAIDs" ... "ESS for matching-adjusted model targeting the ATE were 4,910 exposed and 236,721 controls." |
| **Variables** | 7 | Clearly define all outcomes, exposures, predictors, potential confounders, and effect modifiers. Give diagnostic criteria, if applicable | Methods, Exposure. | "Exposure was defined as dispensation of any NSAID during the first trimester, from the first day of the last menstrual period through the end of the 13th gestational week." ... "exposure was evaluated separately for each NSAID: ibuprofen, diclofenac, etodolac, naproxen, indomethacin, piroxicam, and lornoxicam" ... "Overall exposure was further quantified by the total number of defined daily doses (DDD)...categorized as no exposure (0 DDD), short-term (1–7 DDD), medium-term (8–21 DDD), and long-term (>21 DDD) exposure" ... "MCMs were defined according to the Metropolitan Atlanta Congenital Defects Program (MACDP)" ... "Obesity was defined as any diagnosis in the 278.0-278.4 ICD-9 range, diabetes mellitus as ICD-9 250.* and gestational diabetes mellitus (GDM) as ICD-9 648.00–648.04. Folic acid use was defined as dispensation during the first trimester. Smoking was defined based on self-report or ICD-9 code 305.1." |
| **Data sources/ measurement** | 8* | For each variable of interest, give sources of data and details of methods of assessment (measurement). Describe comparability of assessment methods if there is more than one group | Methods, Dataset Assembly | "The cohort was constructed by linking four databases. Pregnancy and delivery data were obtained from the SUMC Obstetrics and Gynecology Division, and MCMs diagnosed within the first year of life from the SUMC hospitalization database. Malformations identified before elective terminations were manually retrieved from the SUMC Committee for Termination of Pregnancies registry. All diagnoses were confirmed by board-certified specialists and coded using ICD-9. Medication dispensation data, including prescription and over-the-counter NSAIDs with ATC codes and DDD, were extracted from the CHS database. Databases were merged via national ID and hospitalization number to link maternal, neonatal, and termination records" |
| **Bias** | 9 | Describe any efforts to address potential sources of bias | Methods, Bias and Sensitivity Analysis. | "To evaluate the potential impact of exposure misclassification due to over-the-counter ibuprofen purchases not captured in the dispensation database, we conducted a probabilistic sensitivity ("tipping-point") analysis" ... "we performed a sensitivity analysis by excluding pregnancies exposed other analgesic/antipyretic medications from both the exposed and unexposed groups" ... "we conducted another sensitivity analysis redefining first-trimester exposure to include NSAID dispensations initiated within the two weeks preceding the estimated date of conception" ... "we conducted two additional sensitivity analyses: one excluding adjustment for other analgesics, and another restricting adjustment to exposures that preceded NSAID use." |
| **Study size** | 10 | Explain how the study size was arrived at | Methods, Setting and Participants, Results, first paragraph | "The cohort included all pregnancies of women aged 15–45 years insured by Clalit health services (CHS)...at SUMC between 1998 and 2018" ... "A total of 267,301 births and elective terminations occurred at SUMC between 1998 and 2018. Of these, 265,143 pregnancies met eligibility criteria, and 264,858 complete cases were included in the final cohort" |
| **Quantitative variables** | 11 | Explain how quantitative variables were handled in the analyses. If applicable, describe which groupings were chosen and why | Methods, Exposure | "Overall exposure was further quantified by the total number of defined daily doses (DDD) dispensed during the first trimester, categorized as no exposure (0 DDD), short-term (1–7 DDD), medium-term (8–21 DDD), and long-term (>21 DDD) exposure. The corresponding DDDs were 1200 mg for ibuprofen, 100 mg for diclofenac and indomethacin, 400 mg for etodolac, 500 mg for naproxen, 20 mg for piroxicam, and 16 mg for lornoxicam" |
| **Statistical methods** | 12 | (a) Describe all statistical methods, including those used to control for confounding | Methods, Statistical Analysis | "Un matched-adjusted Risk Ratios (RRs) were estimated using Poisson-regression with a logarithmic link function. Marginal (average) effects were derived with G-computation, and robust variance estimates were obtained with cluster-robust heteroskedasticity-consistent ("sandwich") estimator clustered by patient identifier" ... "generalized full matching was applied on the propensity score...targeting the average treatment effect (ATE)" ... "Adjusted RRs were then estimated using G-computation with weighted Poisson-regression and two-way cluster-robust standard errors, clustered on maternal identifier and matching subclass." ... "Covariates accounted for in the final models included maternal age, ethnicity, lack of perinatal care, diabetes, obesity, folic acid supplementation, gravidity, calendar year, smoking, NSAID indication, and exposure to other analgesics or anti pyretics. These were selected a priori by two experts (S.D. and A.A.H.) to block potential confounding via "backdoor" paths." |
|  |  | (b) Describe any methods used to examine subgroups and interactions | Methods, Exposure, Outcomes | "exposure was evaluated separately for each NSAID: ibuprofen, diclofenac, etodolac, naproxen, indomethacin, piroxicam, and lornoxicam" ... "We examined associations between first-trimester NSAID exposure and the overall prevalence of MCMs, as well as system-specific groups: cardiovascular (ICD-9: 745, 746, 747), central nervous system (740, 741, 742, 743), musculoskeletal (754, 755, 756), gastrointestinal (750, 751), and genitourinary malformations (752, 753)" |
|  |  | (c) Explain how missing data were addressed | Methods, Study Design, Table 1 | "Analyses were restricted to pregnancies with complete data on model's covariates and outcomes." Table 1 footnote: "Missing values for fetal sex reflect pregnancy terminations due to suspected fetal malformations, for which fetal sex could not be determined." |
|  |  | (d) Cohort study—If applicable, explain how loss to follow-up was addressed | Methods, Statistical Analysis | "Matching weights were derived from this procedure and incorporated into the outcome model (no units were discarded by the matching)." |
|  |  | (e) Describe any sensitivity analyses | Methods, Bias and Sensitivity Analysis. | "we conducted a probabilistic sensitivity ("tipping-point") analysis...The proportion reclassified ranged from 0% to 3% of the total cohort in 0.05% increments; at each increment, we performed 100 random reallocations and refit the primary matching-adjusted model" ... "we performed a sensitivity analysis by excluding pregnancies exposed other analgesic/antipyretic medications" ... "we conducted another sensitivity analysis redefining first-trimester exposure to include NSAID dispensations initiated within the two weeks preceding the estimated date of conception" ... "one excluding adjustment for other analgesics, and another restricting adjustment to exposures that preceded NSAID use." |
| ***Results*** | | | | |
| **Participants** | 13* | (a) Report numbers of individuals at each stage of study—eg numbers potentially eligible, examined for eligibility, confirmed eligible, included in the study, completing follow-up, and analysed | Results, first paragraph | "A total of 267,301 births and elective terminations occurred at SUMC between 1998 and 2018. Of these, 265,143 pregnancies met eligibility criteria, and 264,858 complete cases were included in the final cohort (Fig 2). Among those, 20,202 pregnancies (7.6%) were exposed to NSAIDs during the first trimester of pregnancy, including ibuprofen (13,627; 5.13%), diclofenac (4,334; 1.63%), naproxen (3,105; 1.17%), etodolac (1,440; 0.54%), indomethacin (287; 0.1%), piroxicam (91; 0.03%), and lornoxicam (62; 0.02%)." |
|  |  | (b) Give reasons for non-participation at each stage | Methods, Setting and Particitpants, Fig 2. | "Pregnancies exposed to established teratogenic drugs (antimetabolites, isotretinoin, and anti-epileptic drugs), multiple gestations, or pregnancies with documented genetic or chromosomal diagnoses were excluded from the study." Fig 2: "Flow diagram of cohort selection and distribution of first-trimester exposure to individual NSAIDs." |
|  |  | (c) Consider use of a flow diagram | Fig 2 | "Fig 2: Flow diagram of cohort selection and distribution of first-trimester exposure to individual NSAIDs." |
| **Descriptive data** | 14* | (a) Give characteristics of study participants (eg demographic, clinical, social) and information on exposures and potential confounders | Restults, third paragraph. | "Table 1 presents a comparison of pregnancy and maternal characteristics between pregnancies exposed and unexposed to NSAIDs during the first trimester. Exposed pregnancies were more often of Bedouin ethnicity (77% vs. 53%, Standardized Mean Difference [SMD] = 0.50), had higher gravidity (median 4 (IQR; 2, 6) vs 3 (IQR; 2, 5)...higher maternal obesity (1.3% vs. 0.5%, SMD = 0.09), and higher folic acid use (45% vs. 14%, SMD = 0.72)." |
|  |  | (b) Indicate number of participants with missing data for each variable of interest | Table 1 | Table 1: Pregnancy age Missing: 50 (exposed), 661 (unexposed). Sex of newborn Missing: 50 (exposed), 1,578 (unexposed). Footnote: "Missing values for fetal sex reflect pregnancy terminations due to suspected fetal malformations, for which fetal sex could not be determined." |
|  |  | (c) Cohort study—Summarise follow-up time (eg, average and total amount) | Methods, Outcome, Table 1 | "MCMs were defined according to the Metropolitan Atlanta Congenital Defects Program (MACDP)" . Table 1: "Pregnancy age, days: 276 (266, 281) vs 275 (266, 281)" |
| **Outcome data** | 15* | Cohort study—Report numbers of outcome events or summary measures over time | Results, Total NSAID Exposure | "Among 20,202 NSAID-exposed pregnancies, 1,651 (8.2%) were diagnosed with MCMs, compared with 16,998 (7.0%) among the 244,656 unexposed pregnancies." ... "Cardiovascular malformations were identified in 885 (4.4%) of exposed and 7,873 (3.2%) of unexposed pregnancies" |
| **Main results** | 16 | (a) Give unadjusted estimates and, if applicable, confounder-adjusted estimates and their precision (eg, 95% confidence interval). Make clear which confounders were adjusted for and why they were included | Methods, Statistical Analysis, Results, Total NSAID Exposure | "NSAID exposure was associated with an increased risk of MCMs in crude analyses (RR 1.18 (95% CI[1.12,1.24])); however, no association was found after matching (matched aRR 1.00 (95% CI [ 0.89,1.11]))" ... "Covariates accounted for in the final models included maternal age, ethnicity, lack of perinatal care, diabetes, obesity, folic acid supplementation, gravidity, calendar year, smoking, NSAID indication, and exposure to other analgesics or anti pyretics. These were selected a priori by two experts (S.D. and A.A.H.) to block potential confounding via "backdoor" paths." |
|  |  | (b) Report category boundaries when continuous variables were categorized | Results, Dose Response analysis | "The prevalence of MCMs was 8.2% among short term NSAID users (1–7 DDDs), 8.6% among medium term users (8–21 DDDs), and 9.3% among long term users (>21 DDDs), compared with 7.0% in the unexposed group." |
|  |  | (c) If relevant, consider translating estimates of relative risk into absolute risk for a meaningful time period | Results, Total NSAID Exposure, Dose Response analysis | "1,651 (8.2%) were diagnosed with MCMs, compared with 16,998 (7.0%)" ... "Cardiovascular malformations were identified in 885 (4.4%) of exposed and 7,873 (3.2%) of unexposed pregnancies" ... "8.2% among short term NSAID users (1–7 DDDs), 8.6% among medium term users (8–21 DDDs), and 9.3% among long term users (>21 DDDs)" |
| **Other analyses** | 17 | Report other analyses done—eg analyses of subgroups and interactions, and sensitivity analyses | Results, Specific NSAID Exposure  Sensitivity Analysis: | "No associations were observed between exposure to any of the NSAIDs and the risk of overall MCMs (ibuprofen: matched aRR = 0.97 (95% CI [0.86–1.09]); diclofenac: matched aRR = 1.02 (95% CI [0.81,1.30]); naproxen: matched aRR = 0.97 (95% CI [0.73,1.29])..." ... "reclassifying 1.3% of the cohort (with an 8.3% major malformation prevalence among reclassified individuals) yielded a non-significant association in the matching-adjusted analysis (aRR = 1.06 (95% CI [0.97,1.15]))" ... "No association was observed between NSAID exposure and major congenital malformations after exclusion of pregnancies exposed to other analgesic or antipyretic medications (aRR = 1.14 (95% CI [0.93 ,1.41]))" ... "Extending NSAID exposure to two weeks before conception yielded similar results (aRR = 1.02 (95% CI [0.90,1.12]))" |
| ***Discussion*** | | | | |
| **Key results** | 18 | Summarise key results with reference to study objectives | Discussion, first paragraph | "In this matched population-based cohort study, including more than 265,000 singleton pregnancies, exposure to NSAIDs during the first trimester was not associated with increased risk of MCMs, both overall and according to specific organ systems. Consistently, no dose-response was observed between cumulative NSAIDs exposure and malformation risk." |
| **Limitations** | 19 | Discuss limitations of the study, taking into account sources of potential bias or imprecision. Discuss both direction and magnitude of any potential bias | Discussion, fourth paragraph | "This study has several limitations. First, as in other pharmacoepidemiologic investigations, exposure to NSAIDs was determined from dispensation records rather than confirmed intake." ... "Second, our dataset did not capture spontaneous abortions. This omission may lead to underestimation of early pregnancy losses potentially related to teratogenic exposures" ... "Third, ibuprofen's over-the-counter availability could lead to minor exposure misclassification." ... "Some variables commonly considered potential confounders, such as socioeconomic status and maternal education, were unavailable in our dataset. If these factors are associated with both NSAID exposure and major congenital malformations, their omission could introduce residual confounding." |
| **Interpretation** | 20 | Give a cautious overall interpretation of results considering objectives, limitations, multiplicity of analyses, results from similar studies, and other relevant evidence | Discussion, fifth paragraph | "In this large, population-based cohort, we found no evidence supporting an association between exposure to NSAIDs during the first trimester and overall major congenital malformations or specific organ system malformations. We believe these findings have significant clinical relevance given current concerns regarding pain and fever treatment during pregnancy; the comprehensive approach of this study provides a reliable assessment of risk with important implications for both clinical practice and future research in maternal-fetal medicine. While the results are reassuring, further research is needed to provide additional confirmation." |
| **Generalisability** | 21 | Discuss the generalisability (external validity) of the study results | Discussion, first paragraph | "The study population comprises approximately 55% women of Bedouin origin. The Israeli Bedouins are one of the largest Bedouin communities worldwide, with others living in Israel, the Middle East, the Arabian Peninsula, and North Africa. The Bedouin are characterized by distinct demographic and social features, including historically semi‑nomadic roots and relatively high rates of consanguinity compared to other Israeli populations. While these characteristics may differ from those in other settings, the findings contribute important data that complement studies from diverse global populations." |
| ***Other information*** | | | | |
| **Funding** | 22 | Give the source of funding and the role of the funders for the present study and, if applicable, for the original study on which the present article is based | Funding stattment | "No funding was provided." |

# 
